# CLA-GWO: A Bio-Inspired Closed-Loop Adaptive Grey Wolf Optimizer for the Traveling Salesman Problem

**DOI:** 10.3390/biomimetics11100708

**Published:** 2026-10-04

**Authors:** Shang Wang, Yajuan Zhang, Xiangyun Kong

**Affiliations:** 1 Engineering Training Center, Beijing Polytechnic University, Beijing 100176, China; zhangyajuan@bpu.edu.cn; 2 Institute of Engineering Technology, University of Science and Technology Beijing, Beijing 100083, China; supposekong@outlook.com

**Keywords:** grey wolf optimizer, traveling salesman problem, catastrophe-driven, diversity detection, self-learning, metaheuristic optimization

## Abstract

The standard Grey Wolf Optimizer (GWO) suffers from rapid population diversity loss when solving the Traveling Salesman Problem (TSP), leading to premature convergence. This paper proposes the Closed-Loop Adaptive Grey Wolf Optimizer (CLA-GWO), which constructs a five-layer closed-loop adaptive framework comprising: (i) discrete GWO evolution, (ii) dual-threshold diversity detection, (iii) three-level adaptive catastrophe, (iv) two-phase recovery, and (v) self-learning feedback control. The algorithm autonomously perceives population homogenization and restores diversity through scale-adaptive intervention. A two-stage parameter optimization strategy, combining the tree-structured Parzen estimator (TPE) with one-factor-at-a-time (OAT) sensitivity analysis, identified the optimal configuration. Component ablation on seven variants revealed that performance is principally driven by 2-opt local search and nearest-neighbor (NN) initialization, while the catastrophe lockout period (CLP) and multi-level catastrophe mechanisms safeguard diversity. Benchmark comparison on 20 TSPLIB instances demonstrated that CLA-GWO achieves competitive or superior performance under the adopted benchmark protocol against eight state-of-the-art algorithms across primary and secondary comparison groups. A Friedman test confirmed that the overall performance difference among the three independent baselines and CLA-GWO is statistically significant. The framework’s perceive–decide–intervene–recover–learn principle suggests a candidate transferable design pattern for other population-based metaheuristics; its current validation is limited to symmetric TSP, and asymmetric or non-Euclidean variants remain to be examined.

## 1. Introduction

### 1.1. Background and Research Landscape

The Traveling Salesman Problem (TSP) is one of the most intensively studied combinatorial optimization problems in computer science and operations research [1]. Given a set of *n* cities and the pairwise distances between them, the objective is to find the shortest Hamiltonian circuit that visits each city exactly once. The TSP is formally classified as NP-hard [2], and its integer programming formulation was established by Miller, Tucker, and Zemlin [3]. Despite its deceptively simple statement, the TSP has profound practical significance: it serves as a benchmark for algorithmic efficiency and underpins numerous real-world applications in logistics, circuit design, genome sequencing, and planetary mission planning. The TSPLIB library [4] provides a standardized set of benchmark instances that has enabled rigorous cross-algorithm comparisons for decades.

Exact methods (such as branch-and-bound and cutting-plane algorithms) can guarantee optimality but exhibit exponential scalability, becoming intractable beyond a few thousand cities. To address this limitation, specialized heuristic solvers have been developed, most notably the Lin–Kernighan heuristic [5] and its advanced implementation, Lin–Kernighan–Helsgaun (LKH) [6], as well as the edge-assembly crossover genetic algorithm (EAX-GA) [7]. While these specialized solvers can find near-optimal or optimal solutions for instances with thousands of cities, they are complex to implement, require domain-specific data structures, and do not generalize easily to problem variants such as the vehicle routing problem (VRP) or the multiple traveling salesman problem (mTSP). Consequently, metaheuristic algorithms, which offer a favorable trade-off between solution quality, implementation simplicity, and adaptability, remain the dominant approach for practitioners [8].

Bio-inspired metaheuristics draw on natural processes to guide search [9]. Among these, swarm intelligence algorithms, inspired by the collective behavior of social animals, have achieved notable success on the TSP. Ant Colony Optimization (ACO), biologically inspired by the discovery of self-organized foraging shortcuts in Argentine ants, has produced a family of effective TSP solvers, including the original Ant System and the Ant Colony System [10]. Other representative approaches include ACO augmented with local search [11] and collective intelligence frameworks [12]. The Grey Wolf Optimizer (GWO) [13] is a more recent swarm intelligence algorithm that mimics the social hierarchy and cooperative hunting behavior of grey wolves. GWO has gained rapid adoption due to its mathematical simplicity, few control parameters, and an innate mechanism for balancing exploration and exploitation through the linearly decaying convergence factor. A comprehensive review of GWO variants and applications can be found in [14].

In parallel with metaheuristic research, Machine Learning (ML) techniques, particularly neural combinatorial optimization and reinforcement learning (RL), have emerged as alternative paradigms for solving the TSP. Bengio et al. [15] provided a methodological survey of ML for combinatorial optimization, and subsequent work [16] extended this perspective to NP-hard problems more broadly. Neural combinatorial optimization with RL [17] and hybrid RL+LKH approaches [18] have demonstrated promising results on small-to-medium instances. However, a systematic evaluation by Liu et al. [19] revealed that neural methods still struggle to match the solution quality of traditional metaheuristics on larger TSP instances, particularly when generalizing to unseen problem sizes. These findings reinforce the continued relevance of metaheuristic approaches for the TSP, especially in scenarios requiring scalability and robustness.

Despite its broad applicability, the standard GWO faces a critical limitation when applied to the TSP: the rapid loss of population diversity during evolution. As the search progresses, the three leading wolves (α, β, δ) converge to similar positions, and the remaining wolves are drawn toward this diminishing leadership region, causing the entire population to homogenize [20]. Once diversity is lost, the algorithm loses its ability to explore new regions and becomes trapped in local optima—a phenomenon known as premature convergence. This limitation is especially severe on the TSP because the permutation-based search space is high-dimensional and rugged; sustained diversity is therefore required to navigate it effectively.

Several lines of research have attempted to mitigate GWO’s diversity loss. The first direction focuses on parameter adaptation: Hou et al. [21] improved GWO using chaotic tent mapping for initialization and a Gaussian-based nonlinear convergence factor, while Saxena et al. [22] introduced a β-chaotic map to enhance population diversity. The second direction involves hybrid strategies, combining GWO with other algorithms or operators (e.g., differential evolution, chaotic search) to compensate for individual weaknesses. The third direction concerns discretization: adapting the continuous GWO position update to combinatorial problem spaces through encoding transformations (e.g., rank-based mapping). More recently, several studies have applied GWO to the TSP through three primary discretization strategies [23]: (i) rank-based or value-to-permutation mapping that converts the continuous GWO position vector into a city permutation; (ii) crossover-based position update that replaces the arithmetic averaging of the three leading wolves with discrete recombination operators such as order crossover (OX) [24] or partially mapped crossover; and (iii) hybrid frameworks that embed local search operators (e.g., 2-opt, 3-opt) within the GWO iteration loop to refine permutation solutions [25]. A comprehensive survey of these discretization strategies is provided in [14]. Beyond TSP-specific discretizations, GWO continues to be improved and applied in other domains. In automated machine learning, Yu et al. [26] proposed a multi-strategy GWO to automate convolutional-neural-network hyperparameter selection, and Lu et al. [27] further integrated reinforcement learning into a dual-mode GWO for the same crop-disease-recognition task. In discrete routing, recent GWO variants have been developed for TSP-based UAV inspection path planning [28], split-delivery vehicle routing [29], and hierarchical open-pit mine inspection scheduling [30]. However, direct experimental comparison with published GWO-TSP variants remains challenging due to the unavailability of source code and substantial heterogeneity in experimental settings across studies. More broadly, the sensitivity of algorithm rankings to evaluation protocols has been emphasized in engineering optimization: Ghiaskar and Taheri [31] showed that adaptive and evolutionary metaheuristics can change ranking under frequency-constrained truss optimization, highlighting the need for reproducible, multi-metric benchmarking under controlled computational budgets. Therefore, rather than include incomplete or unfair GWO-TSP baselines, this paper adopts two complementary evaluation strategies: it compares the proposed optimizer against pure GWO baselines (PureGWO and PureGWO_NN in Section 4.2) to quantify the improvement attributable to the closed-loop adaptive framework, and against state-of-the-art metaheuristics from other algorithm families (Section 4.3) to assess absolute competitive performance. While all of the above approaches have yielded improvements on continuous benchmark functions and some combinatorial problems, they share a common deficiency: they address the symptoms of diversity loss (through initialization perturbation, convergence factor shaping, or local search embedding) but do not establish a closed-loop control system that actively monitors population health and intervenes when diversity degrades. In particular, no existing GWO variant integrates the full pipeline of diversity detection, adaptive intervention, structured recovery, and self-learning feedback into a unified framework.

### 1.2. The CLA-GWO Approach and Biomimetic Rationale

This paper proposes the Closed-Loop Adaptive Grey Wolf Optimizer (CLA-GWO) to fill this gap. Drawing on the biological analogy of natural catastrophes that reset ecosystem diversity, CLA-GWO constructs a five-layer closed-loop adaptive framework atop a discrete GWO search substrate: (i) GWO evolution with order crossover and budgeted 2-opt refinement; (ii) dual-threshold diversity detection; (iii) three-level adaptive catastrophe matching perturbation intensity to convergence severity; (iv) two-phase recovery combining breadth exploration with depth optimization; and (v) self-learning feedback that adjusts detection sensitivity online.

From a biomimetic perspective, CLA-GWO extends the original GWO’s hunting-based metaphor [13] with two complementary biological metaphors that jointly motivate the closed-loop design. The central metaphor is disturbance-driven diversity reset: natural perturbations disrupt a monoculture and open niches for recolonization, and the three-level catastrophe mechanism analogously disrupts a homogenized population to restore structural diversity. The supporting metaphor is closed-loop homeostasis, in which a self-regulating system senses deviations and corrects them; this frames the dual-threshold detection as the sensory component and the self-learning feedback as the corrective controller. These are conceptual metaphors intended to motivate the design rather than literal models of the referenced biological processes, and the algorithmic mapping does not assert mechanistic equivalence. Together, these metaphors instantiate a “Perceive–Decide–Intervene–Recover–Learn” (PDIRL) cycle that represents a systematic application of closed-loop homeostatic control to population-based optimization. Beyond a specific TSP solver, this work demonstrates that closed-loop homeostatic control, a paradigm ubiquitous in biology yet underexplored in metaheuristic design, can be instantiated in a population-based optimizer.

Despite extensive research on diversity preservation in evolutionary algorithms [32], existing approaches predominantly rely on passive monitoring or static rules with fixed parameters, without a closed-loop mechanism that actively perceives population health and adaptively intervenes. In GWO specifically, while Evolutionary Population Dynamics [20] introduced population-level refreshment, it does not establish a complete PDIRL cycle. For escaping local optima, Iterated Local Search (ILS) [33] and its multi-restart variants [34] are well-established paradigms, and chaotic perturbations have been incorporated into GWO variants [22]. However, these strategies typically employ either fixed-intensity perturbation or stochastic drivers, without multi-level adaptive intensity matched to the degree of population stagnation, and without feedback from recovery effectiveness to calibrate future perturbation sensitivity. In the memetic optimization literature [8,35], the coordination between local search and population-level diversity management is typically governed by static schedules or manual parameter tuning, lacking an autonomous mechanism that determines when to trigger intervention, how to match perturbation intensity, and how to learn from intervention outcomes. These three research strands (diversity preservation, adaptive perturbation, and memetic coordination) have progressed in parallel but have not been integrated into a unified closed-loop control system. CLA-GWO addresses this gap by unifying all three within a PDIRL framework.

### 1.3. Main Contributions

The main contributions of this paper are as follows:

1. An emergent closed-loop control framework. The novelty lies not in any individual mechanism but in the emergent, scale-adaptive behavior that arises when the five layers are coupled into a single loop: the integrated system autonomously transitions between diversity maintenance and diversity creation without manual adjustment, as documented in Section 4.4. The five-layer architecture serves as the enabling substrate for this emergent behavior.
2. A dual-threshold diversity detection mechanism that monitors population homogenization at both the leadership tier (narrow-scope, via edge overlap among α/β/δ) and the population level (wide-scope, via pairwise similarity among top-*K* wolves), providing multi-scale diversity assessment.
3. A three-level adaptive catastrophe mechanism with integrated two-phase recovery (TPR) that selects perturbation intensity (from 5% segment reassembly to 30% double-bridge plus shuffle) based on real-time homogenization degree, while the TPR module combines ILS breadth search with Metropolis acceptance and a large-budget recovery 2-opt, achieving severity-matched intervention and efficient post-catastrophe recovery through a unified exploration-then-intensification pipeline.
4. A self-learning feedback control module that dynamically adjusts detection sensitivity based on historical catastrophe effectiveness and imposes adaptive lockout periods to prevent catastrophe chain reactions, enabling autonomous calibration of intervention intensity across different problem scales.
5. Comprehensive experimental validation on 20 TSPLIB instances (n=100–417) demonstrating that CLA-GWO achieves competitive or superior performance under the adopted benchmark protocol against eight state-of-the-art algorithms, with a Friedman test confirming a statistically significant overall difference ($p<10^{-7}$).

We note that closed-loop control and diversity management themselves build on established research directions (Section 1.1). The contribution of this work is therefore not the engineering assembly of existing operators, but the emergent synergy that their closed-loop coupling produces: the integrated system exhibits scale-adaptive behavior that no individual mechanism, and no ablated variant, reproduces. This is substantiated empirically by the non-additive component gains in Section 4.2 and by the autonomous mode transition in Section 4.4, rather than asserted a priori.

### 1.4. Paper Organization

The remainder of this paper is organized as follows. Section 2 describes the CLA-GWO algorithm in detail, including the discrete GWO evolution, dual-threshold detection, three-level catastrophe, TPR recovery, self-learning feedback, and the complete algorithm flow with complexity analysis. Section 3 presents the experimental design, including benchmark instances, parameter configuration, evaluation metrics, and comparison algorithms. Section 4 reports the results and discussion, covering parameter sensitivity analysis, component ablation, state-of-the-art (SOTA) comparison, convergence and catastrophe analysis. Section 5 concludes the paper with a summary of findings and future directions.

## 2. The Proposed Algorithm

**Figure 1:**
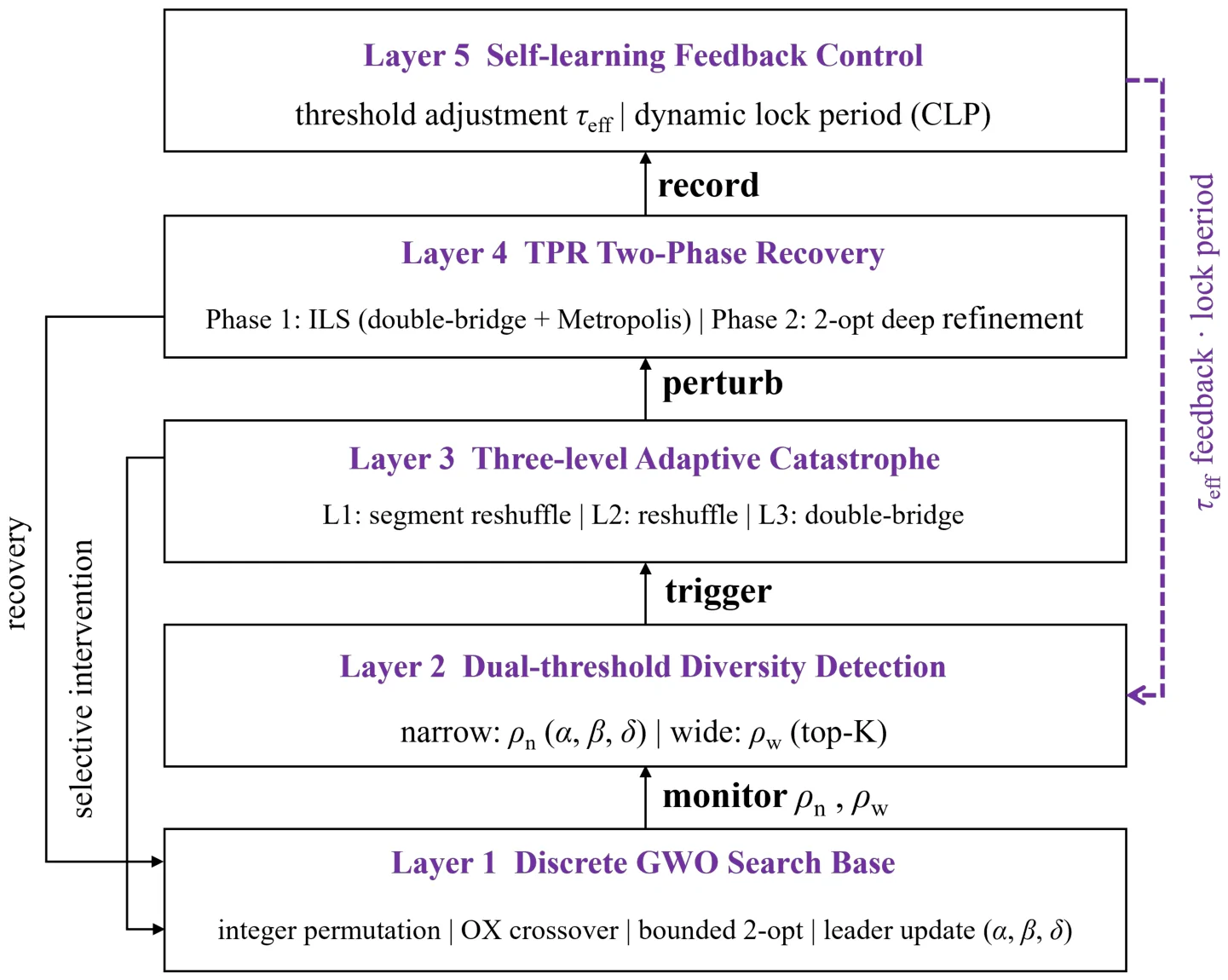
Five-layer closed-loop adaptive control framework of CLA-GWO.

This section presents CLA-GWO in detail. To address the fundamental deficiency of the standard GWO [13,14], namely the rapid loss of population diversity leading to premature convergence when solving the TSP, CLA-GWO constructs a five-layer closed-loop adaptive control system atop a discretized GWO search substrate, following the pipeline of “evolution–detection–catastrophe–recovery–learning.” Specifically, Section 2.1 presents the discrete GWO search framework based on OX; Section 2.2 introduces the dual-threshold diversity detection mechanism; Section 2.3 describes the three-level adaptive catastrophe mechanism; Section 2.4 details the TPR strategy; Section 2.5 presents the self-learning feedback control mechanism; and Section 2.6 provides the complete algorithmic flow and time complexity analysis. The closed-loop interaction among these modules enables real-time perception and active restoration of population health, thereby systematically overcoming the diversity loss problem while preserving the bio-inspired hunting semantics of GWO. The design is grounded in three biological principles (the diversity–stability hypothesis, the intermediate disturbance hypothesis, and biological homeostasis), whose correspondence to algorithmic components is articulated below before the detailed module descriptions. The “Adaptive” in the algorithm name refers to the self-learning feedback control mechanism introduced in Section 2.5.1. Figure 1 illustrates the overall architecture, where each layer’s role is detailed in the corresponding subsection below.

Before detailing the algorithmic components, we articulate the biological principles that motivate the CLA-GWO design. The diversity–stability hypothesis in ecology [36] posits that ecosystems with higher species diversity are more resilient to environmental perturbations, as functional redundancy across species provides insurance against localized failures. The intermediate disturbance hypothesis [37] suggests that perturbations of moderate frequency and intensity sustain the highest diversity. In CLA-GWO, these principles translate into a dual-threshold detection mechanism that monitors population homogenization (analogous to competitive exclusion) and a three-level catastrophe mechanism that applies scale-matched perturbations to restore diversity before stagnation becomes irreversible. The principle of biological homeostasis [38], the self-regulatory process by which living systems maintain internal stability through feedback-driven correction, provides the theoretical foundation for the closed-loop architecture: dual-threshold detection serves as the sensory component, the catastrophe mechanism as the corrective response, and the self-learning feedback module as the sensitivity recalibration layer.

### 2.1. Standard Grey Wolf Optimizer and Its Discretization

#### 2.1.1. Biological Foundation and Mathematical Model of Standard GWO

Grey wolf packs exhibit a strict social hierarchy, comprising α, β, and δ leading wolves and the remaining ω wolves. The hunting process is directed by the leading wolves and followed by the ordinary wolves, exhibiting a three-phase cooperative behavior of “encircling–pursuing–attacking.” The standard GWO [13] abstracts this behavior into the following mathematical model. Let $X_{i}\left(t\right)$ denote the position of the *i*-th wolf at generation *t*, and $X_{p}\left(t\right)$ the position of the prey (current best solution). Then

(1) $$D=|C\circ X_{p}\left(t\right)-X_{i}\left(t\right)|,\;X_{i}\left(t+1\right)=X_{p}\left(t\right)-A\circ D,$$

where ∘ denotes element-wise multiplication, $A=2a\;r_{1}-a$, $C=2r_{2}$, with $r_{1},r_{2}$ being random vectors on [0,1], and the convergence factor *a* linearly decreasing from 2 to 0 over iterations. The three leading wolves each produce a candidate position, and the final position is taken as their average:

(2) $$X_{i}\left(t+1\right)=\frac{X_{\alpha }+X_{\beta }+X_{\delta }}{3}.$$

When |A|>1, the pack expands its search range (global exploration); when |A|<1, the pack converges toward the prey (local exploitation), thereby achieving a dynamic balance between exploration and exploitation.

#### 2.1.2. Challenges of Applying Standard GWO to TSP

The solution space of the TSP is the permutation space of *n* cities, where the encoding of each individual must satisfy permutation validity (each city appears exactly once). The continuous position vectors of the standard GWO do not map directly onto valid permutations; applying rounding or rank-based mapping strategies (e.g., ranked order value or smallest position value) requires additional repair mechanisms, and the geometric semantics of the continuous update formula lose their intended meaning in the discrete domain. More fundamentally, the standard GWO relies solely on elite-guided search within the population, lacking the ability to perceive population health and actively restore diversity [20]. In the later stages of evolution, the population rapidly homogenizes, the search direction converges to a single mode, and the algorithm degenerates into near-random search on TSP instances of moderate scale or larger. Therefore, discretization is merely an adaptation; the fundamental challenge is the mechanistic issue of diversity loss.

#### 2.1.3. Discrete Position Update via Order Crossover

This paper adopts integer permutation encoding, where the position of a wolf is directly expressed as a city permutation $X_{i}=\left(x_{i,1},x_{i,2},\ldots ,x_{i,n}\right)$, with $x_{i,j}\in \left\{1,\ldots ,n\right\}$ and all entries distinct. The continuous-domain notion of “moving toward leading wolves” is discretized into “OX operations using leading wolves as reference parents,” as detailed below.

(1) **Reference parent selection.** The linear decay mechanism of the convergence factor *a* is retained, and $A=2a\;r_{1}-a$ ($r_{1}∼U\left(0,1\right)$) is constructed. Based on the magnitude of |A|, the exploration/exploitation semantics are mapped: when |A|<0.5 (exploitation phase), α, β, and δ are selected as reference parents with probabilities 0.6/0.25/0.15, respectively; when |A|≥0.5 (exploration phase), the selection probabilities are adjusted to 0.4/0.3/0.3 to increase the influence of β and δ, thereby broadening the search directions.

(2) **OX crossover** [24]. For wolf $X_{i}$ and reference parent P, an OX crossover is performed: a contiguous gene segment of the parent is randomly selected and directly inherited by the offspring; the remaining positions are filled in order by the cities of $X_{i}$ not present in the inherited segment, thus ensuring that the offspring is always a valid permutation. This operation preserves the high-quality edge structure of the parent (segment inheritance) and the city ordering information of the other parent (sequential filling), serving as a natural discrete-domain counterpart to “position guidance.”

(3) **Random perturbation.** The offspring undergoes a random segment reversal with a 10% probability to maintain baseline search diversity.

(4) **Elite guidance and budgeted 2-opt refinement.** The three leading wolves are re-identified by fitness each generation; offspring of ordinary wolves are refined by the *evolutionary 2-opt* only when their raw cost is less than 1.8 times the cost of the wolf before the update (i.e., the offspring demonstrate sufficient improvement potential), with a maximum of $I_{\mathrm{gwo}}$ improvements; the three leading wolves undergo mandatory refinement by the top-3 2-opt each generation with a maximum of $I_{\mathrm{top3}}$ improvements. The 2-opt operator employed herein is a candidate-list-based restricted 2-opt (detailed in Section 2.4.2) shared by all usage contexts, which achieves continuous polishing of high-quality solutions through a “budget-constrained, multi-call” strategy with minimal per-call overhead. Throughout the paper, three usage modes of this operator are distinguished by name: evolutionary 2-opt (the conditional refinement of ordinary-wolf offspring in the GWO evolutionary layer), top-3 2-opt (the mandatory per-generation refinement of the leading wolves), and recovery 2-opt (the large-budget deep refinement in the two-phase recovery, Section 2.4.2).

### 2.2. Dual-Threshold Diversity Detection Mechanism

The critical deficiency of the standard GWO lies in its inability to perceive population health, and thus it cannot answer the core question of “when to intervene.” To this end, a dual-threshold diversity detection mechanism is proposed to monitor the degree of population homogenization in real time from two complementary scales.

#### 2.2.1. Narrow-Scope Detection

Define the edge overlap ratio between two tours *X* and *Y* as

(3) $$\rho \left(X,Y\right)=\frac{\left|E(X)\cap E(Y)\right|}{n},$$

where E(·) denotes the undirected edge set of a tour. Narrow-scope detection focuses on the leadership tier, computing the average edge overlap ratio among the three leading wolves α, β, and δ:

(4) $$\rho_{n}=\frac{\rho (\alpha ,\beta )+\rho (\alpha ,\delta )+\rho (\beta ,\delta )}{3}.$$

When the path structures within the leadership tier are highly similar ($\rho_{n}$ approaching 1), the search has entered a local convergence phase: even if other individuals retain diversity, the algorithm has lost the guiding direction for further improvement, necessitating timely intervention.

#### 2.2.2. Wide-Scope Detection

Narrow-scope detection has a blind spot: the overall edge composition of the population may have already become highly homogeneous while the three leading wolves have not yet fully converged. To address this, wide-scope detection selects the top-*K* wolves by fitness and computes their pairwise average edge overlap ratio:

(5) $$\rho_{w}=\frac{2}{K(K-1)}\sum_{1\leq i<j\leq K}\rho \left(t_{i},t_{j}\right),$$

where $t_{i}$ is the tour of the *i*-th top wolf. Wide-scope detection characterizes the overall diversity level of the population, compensating for the blind spot of narrow-scope detection regarding homogenization outside the leadership tier.

#### 2.2.3. Joint Triggering Strategy

Let $\tau_{n}$ and $\tau_{w}$ denote the static detection thresholds for the narrow-scope and wide-scope channels, respectively, and $\tau_{\mathrm{eff}}$ the effective detection threshold output by the self-learning module (Section 2.5). The trigger thresholds for the two channels are

(6) $$\theta_{n}=\max\left(\tau_{\mathrm{eff}},\;\tau_{n}\right),\;\theta_{w}=\max\left(\tau_{\mathrm{eff}},\;\tau_{w}\right).$$

A catastrophe recovery procedure is triggered when $\rho_{n}>\theta_{n}$ or $\rho_{w}>\theta_{w}$. This joint triggering strategy ensures timely response under both scenarios of “local convergence of the leadership tier” and “global diversity loss of the population.” Meanwhile, the maximum homogenization degree $\rho_{\max}=\max\left(\rho_{n},\rho_{w}\right)$ is used for the level decision of catastrophe intensity (see Section 2.3.1). Detection is executed only after the lockout period (Section 2.5.2) has expired, preventing repeated triggers during the recovery phase.

### 2.3. Three-Level Adaptive Catastrophe Mechanism

The core design philosophy of the catastrophe mechanism is selective intervention differentiated by the severity of convergence, rather than indiscriminately disrupting the population.

#### 2.3.1. Catastrophe Intensity Classification

Based on $\rho_{\max}$, the population state is classified into three convergence levels:

(7) $$\mathrm{level}=\left\{\begin{array}{cc} 3, & \rho_{\max}\geq 0.95\;(\mathrm{severe}\;\mathrm{homogenization}),\\ 2, & 0.85\leq \rho_{\max}<0.95\;(\mathrm{moderate}\;\mathrm{convergence}),\\ 1, & 0.70\leq \rho_{\max}<0.85\;(\mathrm{mild}\;\mathrm{convergence}). \end{array}\right.$$

The three levels correspond to increasing perturbation intensities: the mild level disrupts a proportion $r_{d}^{\left(1\right)}=0.05$ of segments, partitioning the tour into several segments and randomly reassembling them; the moderate level increases the disruption proportion to $r_{d}^{\left(2\right)}=0.15$, implementing a larger-scale segment reassembly; the severe level applies a double-bridge perturbation and additionally shuffles 30% of city positions, achieving global restructuring. This forms a seamless progression from micro-perturbation to large-scale restructuring, avoiding the dilemma of fixed-intensity catastrophes between “excessive disruption” and “insufficient recovery.” Permutation repair is performed after the catastrophe operation to ensure tour validity.

#### 2.3.2. Selective Wolf Intervention

Catastrophe operations are applied only to ordinary wolves, excluding the three leading wolves to preserve high-quality solutions. The proportion of wolves involved in the catastrophe at each level is $r_{c}^{\left(l\right)}\in \left\{0.20,0.40,0.60\right\}$ (for levels 1/2/3, respectively), meaning the number of intervened wolves is $\max(1,\left\lfloor N\cdot r_{c}^{\left(l\right)}\right\rfloor )$, randomly selected from the wolves excluding the leaders. The intervention scale adaptively increases with the severity of convergence, balancing disruption efficiency with high-quality solution preservation.

### 2.4. TPR Two-Phase Catastrophe Recovery

If recovery is not promptly performed after catastrophe disruption, the solution quality of the population would deteriorate drastically. To this end, a TPR strategy is proposed, following the principle of exploration-then-intensification: first exploring alternative structures over a larger range, then applying deep refinement to candidate solutions.

#### 2.4.1. Phase 1: ILS Breadth Search

For each intervened wolf, $k_{1}$ rounds of ILS are performed. Each round subjects the current solution to a double-bridge perturbation to escape the local structure, then applies a 2-opt refinement with a maximum of $I_{\max}$ improvements to produce a candidate solution. The candidate is accepted according to the simulated-annealing-style Metropolis criterion:

(8) $$P_{\mathrm{acc}}=\left\{\begin{array}{cc} 1, & \Delta f<0,\\ \exp(-\Delta f/T), & \Delta f\geq 0, \end{array}\right.\;T=0.1\;\bar{f},$$

where Δf is the cost difference between the candidate and current solutions, and $\bar{f}$ is the mean fitness of the current population. This criterion accepts better solutions while accepting worse solutions with an appropriate probability, maintaining search diversity after catastrophe.

#### 2.4.2. Phase 2: Recovery 2-Opt Deep Optimization

Building upon the Phase 1 search results, a large-budget recovery 2-opt refinement is applied to the candidate solutions, with a maximum of $I_{\max}\times k_{2}$ improvements, achieving deep intensification. The 2-opt employed in this paper is not the textbook standard 2-opt [39], but an engineering implementation oriented toward large-scale TSP, featuring three characteristics: (i) restricted candidate neighborhood: only the nearest $c_{\mathrm{cand}}$ neighbors of each edge’s starting endpoint are considered, reducing the neighborhood size from $O(n^{2})$ to $O(n\cdot c_{\mathrm{cand}})$; (ii) first-improvement strategy: the first swap that yields a cost reduction is immediately applied; (iii) incremental evaluation and budget control: position index arrays and a precomputed distance matrix enable O(1) cost evaluation, and each call is constrained by an improvement budget, substituting “multiple distributed calls” for “single-call full convergence.” The sequential order of Phase 1 and Phase 2 ensures the recovery effect of exploration followed by intensification.

### 2.5. Self-Learning Feedback Control

To equip the algorithm with the ability to autonomously adjust its intervention strategy throughout the search process, a self-learning feedback control module is introduced, comprising two components: threshold adjustment and lockout period control.

#### 2.5.1. Self-Learning Threshold Adjustment

The self-learning module records the α cost before each catastrophe, $f_{\mathrm{before}}$, and the best cost after recovery, $f_{\mathrm{after}}$, then computes the catastrophe’s net improvement as $\delta =\max(0,f_{\mathrm{before}}-f_{\mathrm{after}})$. The average improvement magnitude over the most recent *H* catastrophes, $\bar{\delta }$, is computed and normalized by a reference cost to adjust the effective detection threshold:

(9) $$\tau_{\mathrm{eff}}=\tau_{n}\left(1+\eta \;\frac{\bar{\delta }}{f_{\mathrm{ref}}}\right),\;\tau_{\mathrm{eff}}\in \left[0.75,0.98\right],$$

where η is the learning rate and $f_{\mathrm{ref}}$ is the reference cost. When catastrophes yield significant improvement, the algorithm proportionally increases the threshold to reduce detection sensitivity and avoid unnecessary interventions; when improvement is marginal, the threshold remains near the baseline level, maintaining normal intervention frequency. This mechanism achieves closed-loop self-adaptation of detection sensitivity to catastrophe effectiveness.

The truncation interval [0.75,0.98] for $\tau_{\mathrm{eff}}$ serves two purposes: (i) the lower bound 0.75 prevents excessive intervention frequency: if $\tau_{\mathrm{eff}}$ were allowed to drop below 0.75, the detection threshold would become too permissive, triggering catastrophes before the population has sufficiently converged, thereby wasting computational budget on premature interventions; (ii) the upper bound 0.98 prevents over-relaxed detection: if $\tau_{\mathrm{eff}}$ were allowed to exceed 0.98, the algorithm would rarely trigger catastrophes even under severe homogenization, losing the diversity restoration capability that is the core of the closed-loop design. The specific values were selected based on the following rationale: 0.75 corresponds to the lower end of the tree-structured Parzen estimator (TPE) search space for $\tau_{n}$ ([0.75,0.95]), ensuring that the self-learning module does not push the effective threshold below the range validated by parameter optimization; 0.98 provides a 2% safety margin below complete homogenization (ρ=1.0), preserving a minimal trigger probability even under the most conservative adaptation. The sensitivity of these bounds is verified in Section 4.1.4.

#### 2.5.2. Dynamic Lockout Period Control

Upon triggering a catastrophe, a lockout window, the catastrophe lockout period (CLP), is immediately initiated, during which diversity detection and new catastrophe triggers are suspended, providing the population with an adequate recovery and evolution period. Let $d_{\mathrm{fresh}}$ be the population diversity immediately after the catastrophe and $d_{\mathrm{cur}}$ the current diversity. The lockout window is released when either of the following conditions is met:

(10) $$d_{\mathrm{cur}}<d_{\mathrm{fresh}}\times \theta_{l}\left(\mathrm{level}\right)\;\mathrm{or}\;\mathrm{duration}>L_{\max},$$

where $\theta_{l}\left(\mathrm{level}\right)\in \left\{0.7,0.5,0.3\right\}$ is a decay threshold decreasing with catastrophe level, and $L_{\max}$ is the maximum lockout period length. This mechanism prevents catastrophe chain reactions, i.e., population oscillations caused by triggering new catastrophes before the recovery from the previous one is complete.

### 2.6. Complete Algorithm Flow

**Figure 2:**
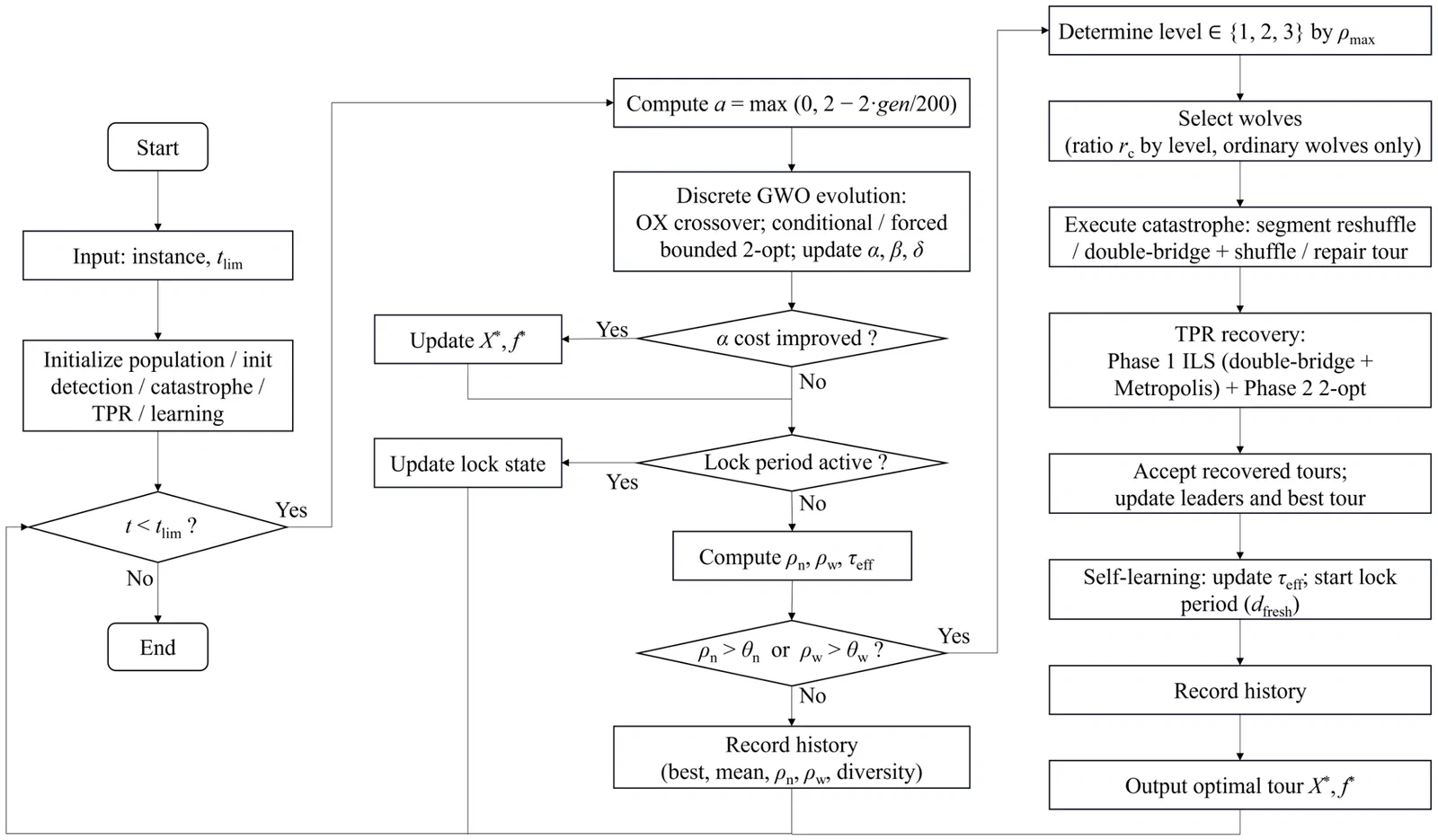
Complete algorithm flow of CLA-GWO.

Figure 2 presents the complete algorithm flow, and Algorithm 1 summarizes the corresponding procedure in pseudocode form. The algorithm initializes the population with one nearest-neighbor (NN) heuristic solution, constructed from a randomly selected start city, plus N−1 random permutations. It then enters a main loop that alternates between GWO evolution and closed-loop control (detection–trigger–catastrophe–recovery–learning). A key design principle is that detection continuously monitors but triggers only after the lockout window has expired, ensuring “recovery before re-intervention.”
**Algorithm 1** Pseudocode of CLA-GWO for the TSP.**Require:** TSP instance *I* with *n* cities; population size *N*; termination criterion (maximum generations *T* or time budget, Section 3.2.3); parameters $\tau_{n}$, $\tau_{w}$, *K*, $c_{\mathrm{cand}}$, $I_{\mathrm{gwo}}$, $I_{\mathrm{top3}}$, $I_{\max}$, $k_{1}$, $k_{2}$, η, *H*, $L_{\max}$, $Gen_{\mathrm{ref}}$**Ensure:** Best tour $\pi^{*}$ and its cost $f^{*}$  1:Initialize population: one nearest-neighbor tour from a randomly selected start city plus (N−1) random permutations  2:Identify leaders {α,β,δ} by fitness; $f^{*}\leftarrow f\left(\alpha \right)$; $\tau_{\mathrm{eff}}\leftarrow \tau_{n}$; CLP inactive  3:**while** termination criterion is not met **do**  4:     $a\leftarrow \max\{0,\;2-2t/Gen_{\mathrm{ref}}\}$  5:     **for** each ordinary wolf $w_{i}\in P\setminus \left\{\alpha ,\beta ,\delta \right\}$ **do**  6:       $A\leftarrow 2a\;r_{1}-a$; select reference parent P∈{α,β,δ} with probabilities 0.6/0.25/0.15 if |A|<0.5, else 0.4/0.3/0.3  7:       $f_{\mathrm{old}}\leftarrow f\left(\pi_{i}\right)$  8:       $\pi_{i}\leftarrow \mathrm{OX}\left(P,\pi_{i}\right)$; random segment reversal with probability 0.1  9:       **if** $f\left(\pi_{i}\right)<1.8\;f_{\mathrm{old}}$ **then** 10:        $\pi_{i}\leftarrow \mathrm{evolutionary}\;\mathrm{2-opt}\left(\pi_{i};\;I_{\mathrm{gwo}},\;c_{\mathrm{cand}}\right)$ 11:     **end if** 12:   **end for** 13:   Re-identify {α,β,δ} by fitness 14:   **for** each leader w∈{α,β,δ} **do** 15:     $w\leftarrow \mathrm{top-3}\;\mathrm{2-opt}(w;\;I_{\mathrm{top3}},\;c_{\mathrm{cand}})$ 16:   **end for** 17:   Update $f^{*}$ 18:   **if** CLP is active **then** 19:     Release CLP if $d_{\mathrm{cur}}<d_{\mathrm{fresh}}\cdot \theta_{l}\left(L\right)$ ($\theta_{l}\in \left\{0.7,0.5,0.3\right\}$ by level) or lockout duration exceeds $L_{\max}$ 20:   **else** 21:     Compute $\rho_{n}$ (leaders) and $\rho_{w}$ (top-*K* wolves); $\theta_{n}\leftarrow \max\left(\tau_{\mathrm{eff}},\tau_{n}\right)$, $\theta_{w}\leftarrow \max\left(\tau_{\mathrm{eff}},\tau_{w}\right)$ 22:     **if** $\rho_{n}>\theta_{n}$$\rho_{w}>\theta_{w}$ **then** 23:        L←3 if $\max(\rho_{n},\rho_{w})\geq 0.95$; 2 if ≥0.85; 1 otherwise (≥0.70) 24:        Select $r_{c}^{\left(L\right)}\cdot N$ ordinary wolves (excluding the leaders) as victims *S*, $r_{c}^{\left(L\right)}\in \left\{0.20,0.40,0.60\right\}$ 25:        Record $f_{\mathrm{before}}$ 26:        **for** each victim wolf w∈S **do** 27:          Apply level-*L* disruption: segment reassembly with ratio $r_{d}^{\left(L\right)}\in \left\{0.05,0.15,0.30\right\}$ (level 3: double-bridge plus 30% city shuffle) 28:          **Phase 1:** $k_{1}$ ILS rounds (double-bridge + 2-opt with budget $I_{\max}$); accept under the Metropolis criterion with $T=0.1\bar{f}$ 29:          **Phase 2:** recovery 2-opt with budget $I_{\max}\times k_{2}$ 30:          Replace *w* if improved 31:        **end for** 32:        Update $f^{*}$; record $f_{\mathrm{after}}$; recompute $\bar{\delta }$ over the most recent *H* catastrophes 33:        $\tau_{\mathrm{eff}}\leftarrow \tau_{n}\left(1+\eta \bar{\delta }/f_{\mathrm{ref}}\right)$, truncated to [0.75,0.98] 34:        Activate CLP with level *L*; record $d_{\mathrm{fresh}}$ 35:     **end if** 36:   **end if** 37:   t←t+1 38:**end while** 39:**return** $\pi^{*},\;f^{*}$

#### Time Complexity Analysis

Let *n* be the number of cities, *N* the population size, $c_{\mathrm{cand}}$ the candidate list size, and *K* the number of wolves for wide-scope detection. The per-generation cost is dominated by three components: (i) GWO evolution, where OX crossover is O(n), conditional 2-opt refinement of ordinary wolves is $O(N\cdot I_{\mathrm{gwo}}\cdot n\cdot c_{\mathrm{cand}})$, and mandatory refinement of leading wolves is $O(3\cdot I_{\mathrm{top3}}\cdot n\cdot c_{\mathrm{cand}})$; (ii) diversity detection, where narrow-scope detection is O(3n) and wide-scope detection is $O(K^{2}n)$; (iii) catastrophe and recovery (executed only on triggered generations), where the catastrophe operation is O(n), TPR Phase 1 is $O(n_{c}\cdot k_{1}\cdot I_{\max}\cdot n\cdot c_{\mathrm{cand}})$, and Phase 2 is $O(n_{c}\cdot k_{2}\cdot I_{\max}\cdot n\cdot c_{\mathrm{cand}})$, where $n_{c}$ is the number of intervened wolves. Since each 2-opt call is constrained by an improvement budget and based on a restricted candidate neighborhood, the per-call cost is significantly lower than the $O(n^{2})$ full-scan of standard 2-opt. The overall algorithm cost is dominated by the budgeted 2-opt calls across all modules and scales linearly with the budget parameters.

The CLA-GWO constructed in this section systematically addresses the diversity loss problem of the standard GWO through a five-layer closed loop of “evolution–detection–catastrophe–recovery–learning”: the discretized search provides a valid and effective evolutionary substrate, the dual-threshold detection answers “when to intervene,” the three-level catastrophe answers “how to intervene,” the TPR recovery answers “how to recover,” and the self-learning feedback answers “how to adaptively adjust the intervention strategy.” The final parameter values of each module are determined through Bayesian hyperparameter optimization and single-factor sensitivity analysis, with the determination process and values detailed in Section 4.1 and the parameter summary table. No formal convergence or diversity-preservation guarantee is provided, because the closed loop operates on a discrete, non-differentiable permutation space with an adaptive, stateful controller (detection, catastrophe, lockout, and self-learning), which makes a rigorous theoretical analysis intractable; the effectiveness of CLA-GWO is therefore established empirically through the controlled experiments and statistical tests in Section 4.

## 3. Experimental Design and Evaluation Metrics

This section describes the experimental design underlying all results reported in Section 4, including the benchmark test instances, parameter configuration, evaluation metrics, and comparison algorithms.

### 3.1. Benchmark Test Set

All experiments are conducted on standard instances from the TSPLIB library [4], a widely used benchmark repository for the TSP. Instance selection follows three principles: (i) scale coverage: instances span from 52 to 417 cities to evaluate scalability; (ii) topological diversity: instances include uniformly distributed points (e.g., kroA100), clustered points (e.g., pr136), and geographically stretched distributions (e.g., pr299); (iii) comparability: selected instances overlap with those reported in the SOTA literature to enable cross-referencing of published results.

Two instance sets are used for different experimental phases:

#### 3.1.1. Parameter Optimization and Ablation Set (Case 4)

Four TSPLIB instances, berlin52 (n=52), rd100 (n=100), kroB150 (n=150), and rat195 (n=195), are selected for parameter optimization (Section 4.1) and component ablation (Section 4.2). These instances cover small, medium, and large scales, and the time budget per instance is set proportional to problem size to ensure comparable convergence levels across scales (Table 1). The published Best Known Solution (BKS) of each instance serves as the reference cost in subsequent performance evaluation.

#### 3.1.2. SOTA Comparison Set (Case 20)

Twenty TSPLIB instances ranging from n=100 to n=417 are used for the SOTA comparison experiments (Section 4.3). These instances are grouped into small (n≤136), medium (150≤n≤200), and large (n>200) categories (Table 2). All 20 instances have published results from at least one SOTA algorithm, enabling cross-referencing.

### 3.2. Parameter Configuration

#### 3.2.1. Parameter Overview

Table 3 presents the complete parameter configuration of CLA-GWO, organized by module. The 8 tunable parameters (marked with ^†^) are determined through the two-stage optimization process described in Section 4.1; the remaining parameters are fixed at values determined by design rationale or supplementary verification.

#### 3.2.2. Tunable vs. Fixed Parameters

The 8 tunable parameters span the four functional layers of CLA-GWO: GWO evolution (*N*), detection ($\tau_{n}$, $\tau_{w}$), TPR recovery ($I_{\max}$, $k_{1}$, $k_{2}$), and self-learning (η, $L_{\max}$). Their search spaces and final values are summarized in Table 3 below. The catastrophe ratios $r_{c}^{\left(l\right)}$ and destruction ratios $r_{d}^{\left(l\right)}$ are fixed at [0.20,0.40,0.60] and [0.05,0.15,0.30], respectively, following the principle of progressively increasing perturbation intensity. The candidate list size $c_{\mathrm{cand}}$ is set to 20, and the wide-scope detection wolf count *K* is set to 10, both determined through supplementary controlled experiments detailed in Section 4.1.4. The convergence-factor reference period $Gen_{\mathrm{ref}}=200$ is fixed rather than tuned because it is a convergence schedule rather than a search operator: it is set to twice the Case-20 generation budget so that the exploration–exploitation factor *a* decays from 2.0 to 1.0 and remains within [1,2] throughout a run, never entering the pure-exploitation regime. Diversity restoration is instead handled by the explicit detection–catastrophe–recovery loop, which operates independently of *a*. A one-factor-at-a-time scan over $Gen_{\mathrm{ref}}$ was therefore omitted, because it would probe only this safety margin rather than the primary performance drivers. The final values of all parameters are determined through Bayesian hyperparameter optimization and single-factor sensitivity analysis, as detailed in Section 4.1.

#### 3.2.3. Experimental Environment and Run Settings

To ensure computational reproducibility, all experiments were implemented in Python 3.13. The computational platform consisted of a workstation featuring an Intel Core i7-7700K processor operating at 4.20 GHz with 16 GB of RAM. In the component ablation experiments (Section 4.2), each test instance was independently solved 20 times with distinct random seeds, whereas the SOTA comparison (Section 4.3) employed 30 independent runs per instance; both settings yield a sufficient sample for statistical analysis of the mean, variance, and best-case performance. A single unified codebase was employed for all algorithm variants, standardizing data structures, random-seed management, and termination conditions. This approach precluded biases that might arise from different software configurations. Distances were computed under two conventions: the SOTA comparison (Section 4.3) uses the TSPLIB integer rounding convention so that tour costs are directly comparable to the published integer BKS values, whereas the relative comparisons (Section 4.1 and Section 4.2) retain floating-point Euclidean distances for finer-grained differentiation. This dual convention is further discussed in Section 3.4.3 and Section 4.5.

Seed management followed a strict isolation protocol across experimental phases to prevent data leakage: the TPE search (Section 4.1.1) used seeds 2000+trial×100+case×10+run; the one-factor-at-a-time (OAT) sensitivity scan (Section 4.1.2) used seeds 3000+case×10+run; the ablation experiments (Section 4.2) used seeds 6000+case×10+run, with shared seeds across variants for paired comparison; the 30-run validation (Section 4.1.3) used seeds 7000+case×100+run (e.g., 7100–7129 for berlin52); and the SOTA comparison (Section 4.3) used seeds 5000+case×100+run. All seed spaces were mutually non-overlapping.

The termination condition for Case 4 instances was a time budget proportional to problem size (Table 1); for Case 20 instances, the maximum generation count was set to 100. The convergence factor *a* decays over a reference period of $Gen_{\mathrm{ref}}=200$ generations (Table 3), meaning that during a 100-generation Case 20 run, *a* decreases from 2.0 to 1.0, maintaining balanced exploration and exploitation throughout without fully transitioning to pure exploitation. This design is intentional: the catastrophe mechanism (Section 2.3) provides diversity injection independent of *a*, ensuring that the algorithm can escape local optima even as *a* decreases. For Case 4 instances terminated by time budget, the actual generation count varied by problem size, and $Gen_{\mathrm{ref}}=200$ served as the same decay reference. The parameters tuned under the time-budget protocol of Case 4 remain appropriate for the generation-budget protocol of Case 20 because all tunable parameters regulate per-generation search behavior (population size, detection sensitivity, and recovery budgets) rather than the termination criterion; the only termination-linked quantity is the convergence schedule $Gen_{\mathrm{ref}}$, which is fixed and budget-independent. The 30-run validation (Section 4.1.3), which imposes the same 100-generation cap as Case 20, further confirms this transferability.

### 3.3. Evaluation Metrics

#### 3.3.1. Optimality Gap

The primary evaluation metric is the optimality gap (Gap), defined as the percentage deviation of the obtained solution cost from the BKS:

(11) $$\mathrm{Gap}\left(\%\right)=\frac{f(X)-\mathrm{BKS}}{\mathrm{BKS}}\times 100,$$

where f(X) is the tour cost of solution X and BKS is the published optimal or best-known cost. Four Gap statistics are reported: **Best Gap** (minimum across all runs), **Mean Gap** (arithmetic mean), **Worst Gap** (maximum), and **Std Gap** (sample standard deviation, ddof = 1). A Gap of 0% indicates that the algorithm has found a solution matching the BKS. For the relative comparisons evaluated in floating-point arithmetic, a best cost may fall marginally below the published integer-valued BKS; in all reported statistics, such negative raw Gap values are clamped to zero, indicating attainment of the BKS level within the floating-point margin rather than an improvement over it. Under the integer convention used for the SOTA comparison, tour costs are integers and this situation does not arise.

#### 3.3.2. Success Rate

The success rate (SR) measures the reliability of the algorithm in achieving near-optimal solutions, defined as the proportion of runs yielding a Gap below a threshold θ:

(12) $$\mathrm{SR}\left(\%\right)=\frac{\left|\{i:{\mathrm{Gap}}_{i}<\theta \}\right|}{N_{\mathrm{run}}}\times 100,$$

where $N_{\mathrm{run}}$ is the total number of independent runs. The threshold is set at θ=1%, consistent with the convention adopted in recent TSP metaheuristic studies.

#### 3.3.3. Aggregate Gap and Degradation

For the ablation analysis (Section 4.2), two aggregate metrics are defined. The **Sum of Gaps** (Sum_Gap) is the total Mean Gap across all test instances:

(13) $$\mathrm{Sum\_Gap}=\sum_{i=1}^{m}\mathrm{Mean}\;{\mathrm{Gap}}_{i},$$

where *m* is the number of instances. The **degradation** (ΔSum_Gap) measures the performance loss of an ablation variant relative to the Full algorithm:

(14) $$\Delta \mathrm{Sum\_Gap}={\mathrm{Sum\_Gap}}_{\mathrm{variant}}-{\mathrm{Sum\_Gap}}_{\mathrm{Full}},$$

where a positive value indicates degradation (i.e., the removed component contributes positively to performance).

#### 3.3.4. Statistical Tests

To assess the statistical significance of performance differences among multiple algorithms, the following non-parametric tests are employed:

**Friedman test.** The Friedman test [40] ranks algorithms on each instance (1= best, k= worst, where *k* is the number of algorithms) and tests the null hypothesis that all algorithms perform equivalently. The test statistic is:

(15) $$\chi_{F}^{2}=\frac{12n}{k(k+1)}\sum_{j=1}^{k}{\left({\bar{R}}_{j}-\frac{k+1}{2}\right)}^{2},$$

where *n* is the number of instances, *k* is the number of algorithms, and ${\bar{R}}_{j}$ is the average rank of algorithm *j*. A *p*-value below 0.05 indicates a significant difference among algorithms.

**Nemenyi post-hoc test.** When the Friedman test rejects the null hypothesis, the Nemenyi test identifies which pairs of algorithms differ significantly. Two algorithms are significantly different if the difference in their average ranks exceeds the critical difference (CD):

(16) $$\mathrm{CD}=q_{\alpha }\sqrt{\frac{k(k+1)}{6n}},$$

where $q_{\alpha }$ is the critical value from the Studentized range distribution.

**Win/Tie/Loss analysis.** For each pair of algorithms, a pairwise comparison counts the number of instances where one algorithm outperforms (Win), matches (Tie), or underperforms (Loss) the other, providing an intuitive complement to the rank-based tests.

### 3.4. Comparison Algorithms

The SOTA comparison is organized into two groups, following the two-group evaluation paradigm established in [41].

#### 3.4.1. Primary Group

The primary group comprises four widely referenced metaheuristic algorithms:

- **ABCSS** [42]: Artificial Bee Colony with Scatter Search, a hybrid swarm intelligence approach.
- **DSMO** [43]: Discrete Spider Monkey Optimization, adapted for combinatorial problems.
- **DWHO** [44]: Discrete Wild Horse Optimizer, a nature-inspired metaheuristic.
- **GACS** [41]: Globally Adaptive Ant Colony System, a recently published ACO variant with greedy initialization; this algorithm is a prior work of the authors’ research group.

All four algorithms have published results on nearly all of the 20 TSPLIB instances used in this study; the only exception is kroC100, for which ABCSS and DSMO results are unavailable. The comparison data are drawn directly from the original publications.

#### 3.4.2. Secondary Group

The secondary group comprises four recent metaheuristics, together with GACS for cross-group referencing:

- **DDCS** [45]: Discrete Differential Cuckoo Search.
- **AACO-LST** [46]: Adaptive Ant Colony Optimization with Local Search Tabu.
- **D-SFOA** [47]: Discrete Salp Fruit-fly Optimization Algorithm.
- **PM** [12]: the ant-system-based collective intelligence method of Kiran and Beskirli, which employs a pheromone-footprint mechanism (abbreviated PM for “Proposed Method” in the original publication).
- **GACS** [41]: included in both groups for cross-reference.

Due to limited instance overlap in the original publications, the secondary-group comparison is conducted on 7 shared instances (kroA100, kroB100, kroC100, lin105, pr107, kroA150, kroB150).

#### 3.4.3. Fairness Considerations

To ensure a fair and transparent comparison, the following measures are adopted:

1. CLA-GWO is tested exclusively on its own data; all SOTA algorithm results are cited from their original publications, not re-implemented.
2. All compared instances are standard TSPLIB instances with publicly verifiable BKS values.
3. The per-instance runtime of CLA-GWO remains at the same order of magnitude as the computation times reported in the SOTA literature; no algorithm is given disproportionate computational advantage.
4. Specialized TSP solvers (e.g., Concorde, LKH) are not included as competitors, as they operate under fundamentally different paradigms; their published BKS values serve only as reference benchmarks; a dedicated reference comparison with three such solvers is presented in Section 4.3.5.

This cross-study comparison approach follows standard practice in the TSP metaheuristic literature [8]; the two-group design provides multiple reference points per instance to partially mitigate environmental variance.

Table 4 summarizes the computational environment and comparison methodology for each algorithm.

**Per-instance runtime by instance scale.** Table 5 summarizes the per-instance runtime of CLA-GWO by instance scale; under the fixed 100-generation termination rule of the SOTA comparison set, the runtime grows smoothly with problem size *n*.

**Distance calculation convention.** Distances follow the dual convention defined in Section 3.2.3: the SOTA comparison uses the TSPLIB integer (nint) convention, whereas the relative comparisons retain floating-point Euclidean distances. The focused diagnosis on eil101 (Section 4.5) confirmed that the algorithm finds the optimal tour (629) under both conventions.

**Cross-study comparison caveat.** Because all SOTA results are drawn from original publications rather than re-implemented under a unified framework, residual environmental variance (hardware, compiler, distance convention) cannot be fully eliminated. This is a standard limitation in the TSP metaheuristic comparison literature and is acknowledged transparently.

## 4. Results and Discussion

### 4.1. Parameter Sensitivity Analysis

The performance of metaheuristic algorithms is highly sensitive to parameter selection. To avoid the subjective bias introduced by manual parameter tuning, this study adopts a two-stage parameter optimization strategy: the first stage (this subsection) employs TPE [48] for global coarse search to identify key parameters, and the second stage (Section 4.1.2) performs single-factor fine-grained scans to verify the stability of optimal values.

#### 4.1.1. TPE-Based Hyperparameter Search

The search space encompasses 8 parameters, namely 5 discrete (N∈{20,30,50}, $k_{1}\in \left\{1,3,5\right\}$, $k_{2}\in \left\{3,5,8\right\}$, $I_{\max}\in \left\{20,30,50\right\}$, $L_{\max}\in \left\{5,10,15\right\}$) and 3 continuous ($\tau_{n}\in \left[0.75,0.95\right]$, $\tau_{w}\in \left[0.55,0.80\right]$, η∈[0.05,0.25]). Experiments were conducted on 4 TSPLIB instances (berlin52, rd100, kroB150, rat195), each configuration independently executed 10 times, with the mean Gap across 4 instances as the loss function. TPE was run for 100 trials (4000 algorithm invocations), with time budgets proportional to problem size (3–18 s).

Table 6 summarizes the overall statistics. The best configuration achieved a mean Gap of 0.5557%; the worst, 1.0560%; and the overall mean was 0.7645±0.1012%. 88% of trials yielded Gap values between 0.6% and 0.9%, indicating reasonable parameter space design and strong robustness. The best configuration (Trial 65) exhibited scale-dependent performance: it achieved near-optimal results on berlin52 (0.005%) but the worst performance on rat195 (1.549%). All Top-5 configurations shared N=20 and $\tau_{n}$ in the 0.76–0.84 range, confirming TPE convergence to a stable optimal region.

To quantitatively identify sensitive parameters, the 100 configurations were ranked by Gap, and the Top-10 and Bottom-10 groups were compared (Table 7).

Four parameters exert significant influence: *N* (−18.0, by far the largest: a smaller population favors rapid convergence under fixed time budgets; N=20 appeared in 62 of 100 trials with the lowest mean Gap of 0.733%); $I_{\max}$ (+3.0: a larger 2-opt budget enhances quality, consistent with its role as the core local search component); $L_{\max}$ (+2.0: a longer lockout period allows thorough post-catastrophe search); and $\tau_{n}$ (−0.0366: a lower threshold enables faster diversity intervention). The remaining 4 parameters ($k_{1}$, $k_{2}$, η, $\tau_{w}$) show small or inconsistent differences and are fixed at Trial 65 values. Table 8 presents the complete optimal configuration, which serves as the baseline for all subsequent experiments.

#### 4.1.2. One-Factor-at-a-Time (OAT) Sensitivity Scan

**Figure 3:**
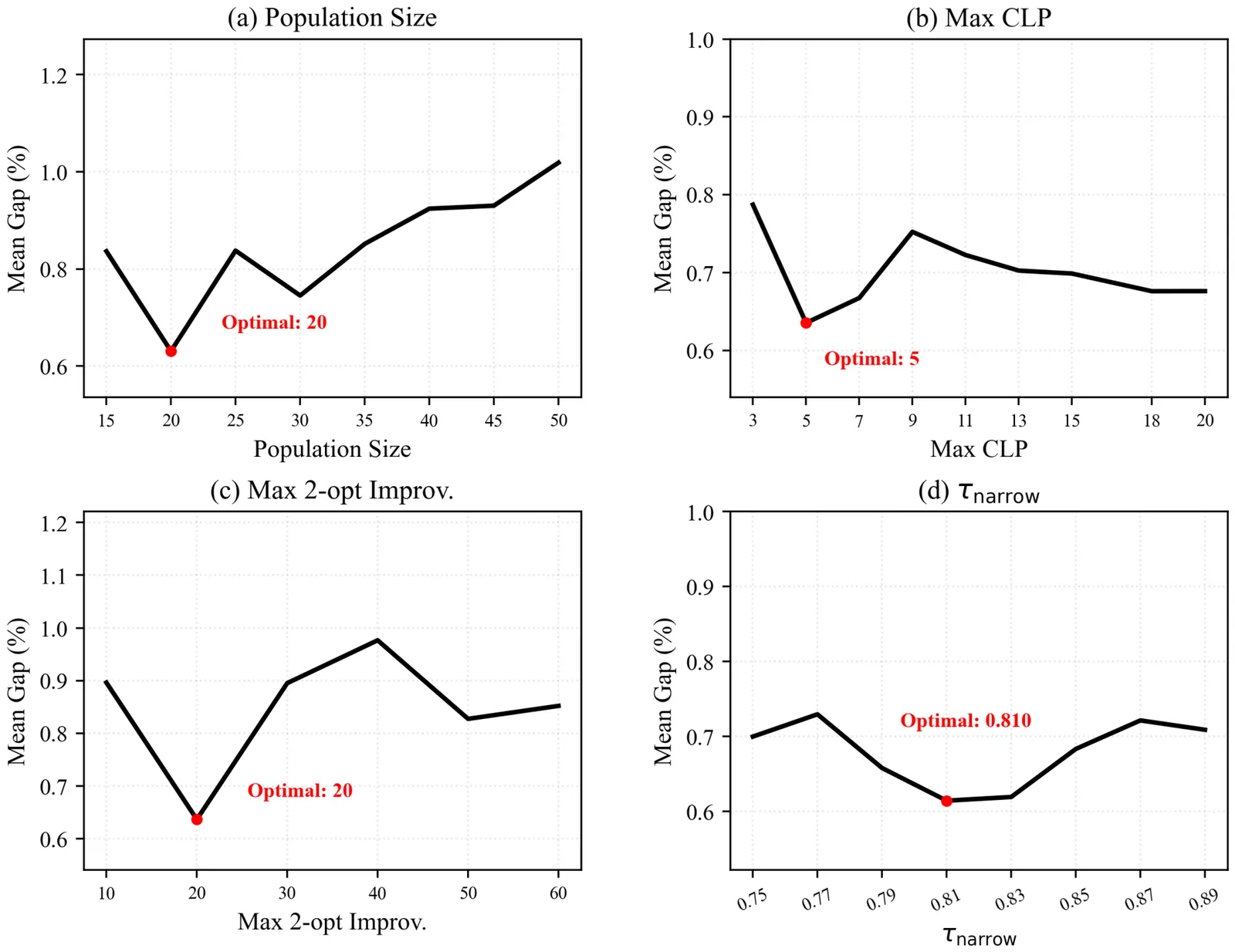
Overall sensitivity curves for the four key parameters: (**a**) population size *N*; (**b**) maximum lockout period $L_{\max}$; (**c**) maximum 2-opt budget $I_{\max}$; (**d**) narrow-scope threshold $\tau_{n}$. Red • marks the scan optimum.

Using the Trial 65 configuration as baseline, OAT scans were conducted on the 4 key parameters: each parameter was varied while others were held fixed, tested on 4 instances with 10 independent runs each. Figure 3 presents the overall sensitivity curves, and the detailed numerical data are provided in Table 9.

**Figure 4:**
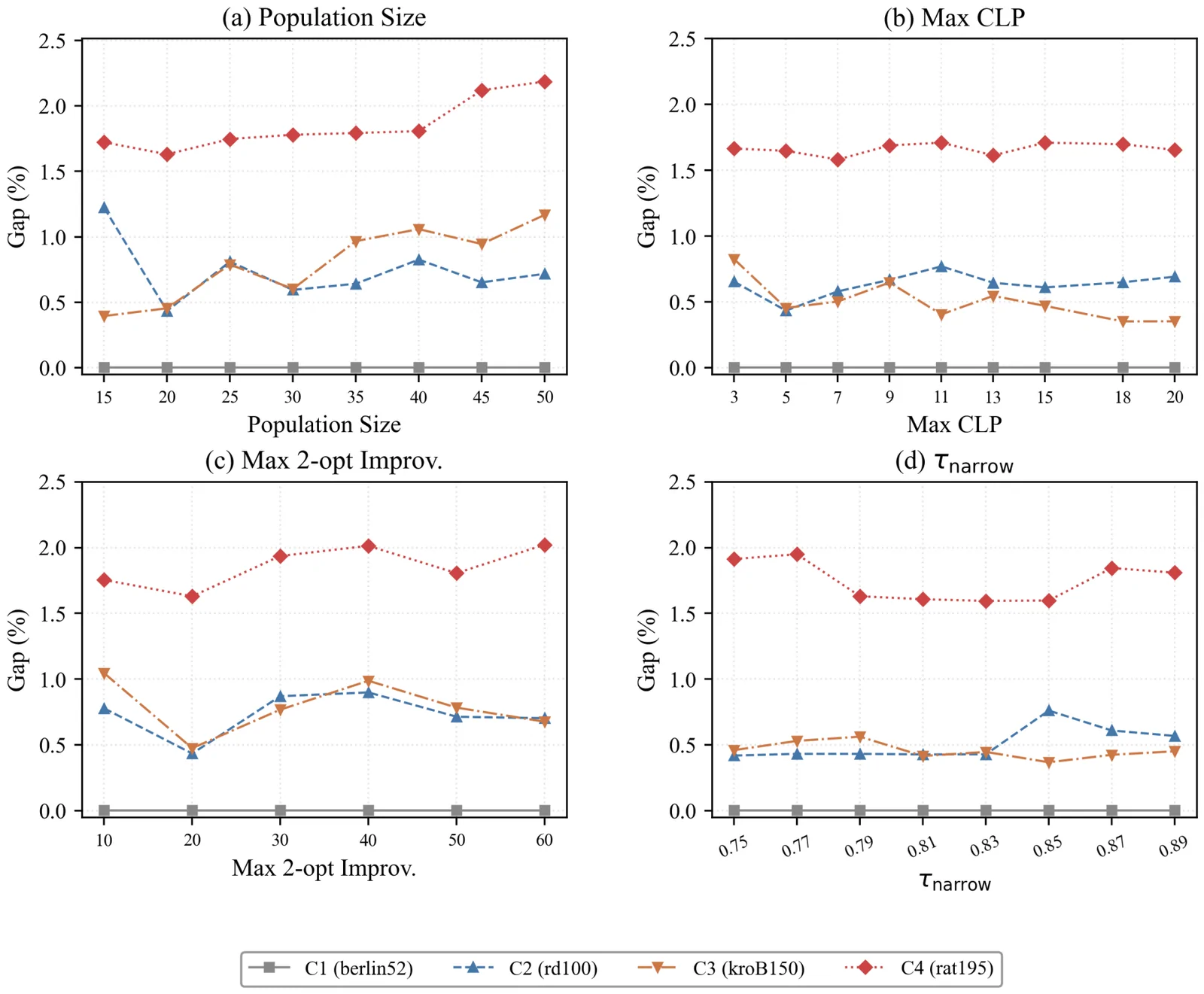
Per-instance sensitivity curves for the four key parameters: (**a**) *N*; (**b**) $L_{\max}$; (**c**) $I_{\max}$; (**d**) $\tau_{n}$.

*N* **(population size).** As shown in Figure 3a, N=20 is optimal (0.631%) with cliff-like deterioration above N=25. The per-instance view (Figure 4a) reveals that rat195 is the primary driver of this deterioration, worsening to 2.19% at N=50, while berlin52 remains insensitive throughout. Consistent with the TPE search where all Top-10 adopted N=20, N=20 is selected.

$L_{\max}$ **(maximum lockout period).** Figure 3b shows $L_{\max}=5$ as optimal (0.635%) with a wide flat plateau in the 7–20 range (0.667–0.752%). $L_{\max}=3$ produces a higher Gap (0.787%), primarily on kroB150. $L_{\max}=5$ is selected, consistent with Trial 65.

$I_{\max}$ **(2-opt improvement budget).** The curve exhibits a clear U-shape (Figure 3c) with the minimum at $I_{\max}=20$ (0.637%). The TPE analysis (Section 4.1.1, Table 9) identified $I_{\max}$ direction as “Higher → Better” because TPE measures the correlation between parameter magnitude and aggregate loss across multi-parameter configurations; in contrast, the OAT scan isolates the marginal effect of $I_{\max}$ alone, revealing that increasing per-call 2-opt iterations beyond 20 linearly raises per-generation cost, reduces total generations achievable within the time budget, and encroaches on the diversity maintenance budget. This counter-intuitive result (more iterations do not improve quality) arises because the two stages measure different dimensions: TPE captures interaction effects among co-varied parameters, while OAT captures the standalone marginal effect. $I_{\max}=20$ is selected.

$\tau_{n}$ **(narrow-scope threshold).** This parameter exhibits the flattest curve (Figure 3d): 0.81 and 0.83 achieve the lowest Gaps (0.614% and 0.619%), with the 0.79–0.83 range varying by only 0.044%. The per-instance view (Figure 4d) confirms this flatness holds across all four instances, indicating strong tolerance to missetting. The Trial 65 value $\tau_{n}=0.800$ falls within this flat region and is selected.

In summary, *N* and $I_{\max}$ exhibit sharp sensitivity profiles (performance deteriorates rapidly away from the optimal value), while $L_{\max}$ and $\tau_{n}$ display wide flat plateaus. The per-instance analysis (Figure 4) further confirms that these flat regions hold consistently across different problem scales, validating the robustness of the final parameter choices. The apparent discrepancy between the TPE-identified direction and the OAT marginal profile is therefore not a conflict but a difference in what each stage measures; the final configuration selects the OAT-confirmed values and remains robust across the four test scales, as further confirmed by the 30-run independent reproduction in Section 4.1.3.

#### 4.1.3. Final Parameter Configuration Determination

The final configuration combines the 4 TPE-identified key parameters (N=20, $L_{\max}=5$, $I_{\max}=20$, $\tau_{n}=0.800$) with the 4 non-sensitive parameters fixed at Trial 65 values (Table 8). To validate stability, 30 independent runs were conducted on each of the 4 instances using seeds 7000+case×100+run (e.g., 7100–7129 for berlin52) fully non-overlapping with prior experiments (Table 10).

On instances with 52–150 cities, the mean Gap remains below 0.7% and the success rate is at least 77%. On berlin52, the search essentially converges to the known optimum (Gap ≈0.005%, std ≈0), and kroB150 achieves 90% SR. On rat195 (n=195), the mean Gap rises to 1.753% (SR =13%), indicating that large-scale performance remains suboptimal, a phenomenon further analyzed in Section 4.5. Overall, the 30-run validation confirms a stable and reproducible configuration (Sum_Gap =2.903%, average SR =70%), serving as the unified baseline for subsequent ablation (Section 4.2) and SOTA comparison (Section 4.3) experiments.

#### 4.1.4. Supplementary Verification of Fixed Parameters

The candidate list size $c_{\mathrm{cand}}$, the 2-opt trigger threshold, and the $\tau_{\mathrm{eff}}$ truncation bounds were fixed at empirically set values rather than optimized through TPE/OAT. To provide formal justification for these choices, supplementary controlled experiments were conducted under a unified protocol: three representative instances (kroA100, kroB150, rat195), each independently run 10 times per configuration with a cap of 100 generations; all configurations within each experiment share identical parameters except the one under investigation. The three experiments below report the candidate list size, the 2-opt trigger threshold, and the $\tau_{\mathrm{eff}}$ truncation bounds, respectively.

**Candidate list size $c_{\mathrm{cand}}$.** Table 11 compares $c_{\mathrm{cand}}\in \left\{5,10,15,20\right\}$. On kroA100, all values achieve the BKS (Gap =0%). On kroB150, $c_{\mathrm{cand}}=20$ yields the lowest mean Gap (0.184% vs. 0.209–0.423% for smaller lists) and the lowest best Gap (0.000%). On rat195, $c_{\mathrm{cand}}=15$ and 20 are nearly tied (1.403% vs. 1.412%), both outperforming $c_{\mathrm{cand}}=5$ (1.584%). $c_{\mathrm{cand}}=20$ is confirmed as the optimal or near-optimal choice across all three scales.

**2-opt trigger threshold.** The threshold determines whether a wolf’s offspring warrant 2-opt refinement: refinement is triggered only when their raw cost is below the parent’s cost multiplied by the threshold. Table 12 compares thresholds {1.5,1.8,2.0,2.5}. A lower multiplier (e.g., 1.5) applies stricter filtering, conserving 2-opt budget but potentially missing refinement opportunities; a higher multiplier (e.g., 2.5) triggers refinement more liberally, risking budget dilution. No single threshold dominates across all instances: 1.8 is optimal on kroA100 (0.000%), 1.5 on kroB150 (0.129%), and 2.0 on rat195 (1.227%). However, the spread across thresholds is narrow, at most 0.35 percentage points (pp) on rat195 and 0.28 pp on kroB150, and 1.8 provides a robust compromise: it achieves the best result on kroA100 and remains within the range of variation on the other two instances.

$\tau_{\mathrm{eff}}$ **truncation bounds.** Table 13 compares four bound configurations. The current setting [0.75,0.98] achieves the lowest mean Gap on kroB150 (0.354%) and near-optimal on the other two instances. Performance sensitivity to the bounds is low: the maximum spread across all configurations is 0.28 pp (rat195: 1.068–1.352%), confirming that the [0.75,0.98] interval is robust and its specific values are not critical to algorithmic performance. Because the truncation bounds define the admissible operating range of the effective threshold, they regulate how readily catastrophes are triggered; the four compared configurations span a meaningful portion of this operating window, and the low performance sensitivity (at most 0.28 pp) indicates that this variation in the triggering regime has little effect on solution quality.

To complement the performance-oriented comparison, the trigger statistics of the same four bound configurations were also recorded on the three instances under per-instance wall-clock budgets of 5, 10, and 15 s, with 10 paired runs per configuration. The average number of catastrophe triggers per run is essentially invariant across configurations, at approximately 10.1, 12.6, and 14.7 on kroA100, kroB150, and rat195, respectively; the only visible change is that tightening the upper bound to 0.95 reduces the kroA100 average from 10.1 to 9.4. The level distribution is likewise stable, with level 3 dominating (about 7.8, 8.9, and 9.3 triggers), level 2 next (about 1.9, 3.0, and 4.4), and level 1 least frequent (at most 1.0). Because the tested lower bounds never exceed the nominal threshold of 0.80, they do not constrain the effective threshold, leaving the upper bound as the operative control; varying that bound changes neither the trigger frequency nor the final gap materially. Together with the small performance spread reported above, this confirms that the empirical 0.75–0.98 interval acts as a safe clamping range rather than a delicate tuning target.

**Note on distance convention.** Consistent with the dual convention defined in Section 3.2.3, the sensitivity and ablation experiments retain floating-point Euclidean distances; because all configurations within each table share this convention, the relative comparisons remain valid.

### 4.2. Component Ablation Analysis

#### 4.2.1. Ablation Design and Variants

To quantitatively evaluate the individual contribution of each key component within the CLA-GWO framework, component ablation experiments are designed and conducted in this section. Using the final parameter configuration (Table 8, Section 4.1.1) as the baseline, specific components are selectively disabled, and each variant is independently executed 20 times on each of the 4 TSPLIB instances, with shared seeds across variants to ensure fair comparison. The evaluation metric is the mean Gap, and the performance difference relative to the Full algorithm (ΔSum_Gap) quantifies each component’s contribution. The Full baseline data are drawn from the 30-run independent validation (Table 10, Sum_Gap =2.903%).

The ablation variants and two pure-GWO baselines are defined as follows:

- **NoTPR**: disable TPR two-phase recovery; after catastrophe, directly execute recovery 2-opt deep optimization.
- **NoCLP**: disable dynamic lockout period control; a new catastrophe may be triggered immediately after the previous one.
- **NoMulti**: disable multi-level catastrophe; fix catastrophe intensity to level 2 (moderate).
- **No2opt**: disable global 2-opt local search; rely solely on the GWO OX crossover operator.
- **PureGWO_NN**: pure GWO with one NN heuristic initial solution + N−1 random permutations, no 2-opt, no CLA components.
- **PureGWO**: pure GWO with all-random initialization, no 2-opt, no CLA components.

The results for all 7 variants are presented in Table 14.

To move beyond percentage-point differences alone, a paired Wilcoxon signed-rank test (two-tailed) with 20 paired runs was applied to each ablation variant against Full on every instance using the shared seeds. The results are summarized in Table 15.

The core search components are decisively significant: No2opt and the two pure-GWO baselines differ from Full at p<0.001 on all four instances, with a Wilcoxon statistic of zero in every paired comparison. For the three diversity-safeguard components (NoTPR, NoCLP, NoMulti), the differences are significant on rd100 and kroB150 (p<0.001); NoCLP is near-significant on rat195 (p=0.0532). berlin52 is excluded from the component-level interpretation because its Full gap (about 0.005%) leaves negligible room for improvement. All p-values are uncorrected for multiple comparisons; the core-component conclusions remain significant under any conventional adjustment.

#### 4.2.2. Dynamic Lockout Period (NoCLP)

NoCLP produces the largest aggregate Sum_Gap degradation (ΔSum_Gap =+0.476%) among the three CLA ablation variants. The degradation is consistent across the three medium-to-large instances: rd100 rises from 0.602% to 0.749%, kroB150 from 0.543% to 0.781%, and rat195 from 1.753% to 1.844%; no instance shows improvement. A paired Wilcoxon test confirms this degradation on rd100 and kroB150 (p<0.001) and a near-significant effect on rat195 (p=0.0532). Furthermore, the standard deviation on kroB150 increases by a factor of approximately 1.8 compared to Full, indicating a pronounced stability degradation. The core mechanism of the CLP is to impose an adaptive lockout window after each catastrophe trigger, during which new catastrophes are suppressed, providing the population with sufficient evolution time for structural recovery. Removing CLP allows catastrophes to fire in rapid succession before the population has recovered (a catastrophe chain reaction), causing diversity to be repeatedly disrupted without adequate restoration and driving the search into inefficient oscillation.

#### 4.2.3. Multi-Level Catastrophe (NoMulti)

NoMulti yields ΔSum_Gap =+0.414%, with the most significant per-instance degradation on rd100 (0.602%→0.917%, +0.315%). Although the mean Gap degradation on kroB150 is moderate (+0.101%), its standard deviation increases by a factor of approximately 2.3—the worst stability degradation among all ablation variants. A paired Wilcoxon test confirms the degradation on rd100 and kroB150 (p<0.001); the rat195 difference is not significant (p=0.927). The multi-level catastrophe mechanism selects among three perturbation intensities (5%, 15%, and 30% segment disruption) based on the real-time homogenization degree ($\rho_{n}$ and $\rho_{w}$). When this mechanism is disabled (fixed at level 2), the fixed moderate perturbation faces a dilemma: for scenarios where a mild perturbation would suffice to escape a local optimum, level 2 is too destructive, breaking already-converged high-quality edges; for scenarios requiring stronger disruption, level 2 is insufficient to introduce enough new edges. The 2.3× standard deviation inflation on kroB150 confirms that single-intensity catastrophe is unreliable in practice: the randomness of search behavior increases markedly, and the performance spread across independent runs widens.

#### 4.2.4. TPR Two-Phase Recovery (NoTPR)

NoTPR yields ΔSum_Gap =+0.207%, confirming that TPR contributes a positive but auxiliary improvement. The degradation is concentrated on medium-scale instances: rd100 rises from 0.602% to 0.750% (+0.148%) and kroB150 from 0.543% to 0.655% (+0.112%); rat195 slightly improves (1.753%→1.700%, −0.053%); berlin52 is unaffected. A paired Wilcoxon test confirms the degradation on rd100 and kroB150 (p<0.001); the rat195 and berlin52 differences are not significant. This diverging pattern indicates that the TPR Phase 1 (ILS breadth search with $k_{1}=1$, double-bridge perturbation, and Metropolis acceptance) provides additional structural diversity after catastrophe, preventing premature convergence of the direct recovery 2-opt, a benefit most pronounced on medium-scale instances. On rat195, the perturbation becomes partially redundant with 2-opt deep refinement, occasionally causing a minor negative impact on final solution quality. Overall, TPR’s independent contribution (ΔSum_Gap =+0.207%), while smaller than CLP and multi-level catastrophe, is consistent in direction and reproducible, validating the importance of the recovery stage in the disruption–recovery closed loop.

#### 4.2.5. 2-Opt Local Search (No2opt)

No2opt produces severe degradation across all instances (ΔSum_Gap =+76.362%; per-instance Gaps range from 14.9% to 26.1%). This result is expected: the OX crossover operator of GWO cannot independently perform effective local refinement in the high-dimensional permutation space of TSP and must rely on 2-opt to translate coarse global search directions into high-quality solutions. This variant effectively serves as a positive control for the ablation framework: if No2opt were to perform comparably to Full, the experimental design itself would be questionable.

#### 4.2.6. Pure-GWO Baselines and the NN Initialization Effect

The PureGWO baseline (all-random initialization, no 2-opt, no CLA) achieves a mean Gap of 74.33% across instances—with kroB150 and rat195 exceeding 96%, rendering the algorithm completely non-functional on medium-to-large TSP instances. Introducing a single NN initial solution (PureGWO_NN) reduces the average Gap from 74.33% to 18.81%, a 55.52 pp improvement (per-instance average; the corresponding Sum_Gap gain is 222 pp). This gain is consistent across all instances and monotonically increases with problem size: berlin52 (+22.04 pp), rd100 (+48.08 pp), kroB150 (+72.92 pp), rat195 (+79.05 pp). The underlying mechanism is the quality-edge propagation capability of the OX crossover: the high-quality edges contained in the NN solution are transmitted to other wolves through crossover selection over generations, progressively biasing the entire population’s search direction. With purely random initialization, the crossover operator lacks any prior structural information, making it unable to accumulate useful edge knowledge within a reasonable timeframe.

#### 4.2.7. PureGWO_NN vs. No2opt: A Critical Finding

A critical observation is that PureGWO_NN (Sum_Gap =75.246%) and No2opt (Sum_Gap =79.265%) achieve nearly identical overall performance, with PureGWO_NN even slightly outperforming No2opt by approximately 4 pp. This reveals an important insight: removing the 2-opt operator isolates the exploration-only contribution, and it shows that the detection–catastrophe–recovery mechanisms alone match, but do not exceed, a pure GWO initialized with a single NN solution. Although No2opt retains all CLA components (dual-threshold detection, three-level catastrophe, TPR recovery, self-learning threshold adjustment, CLP control), its post-catastrophe reconstruction relies on the same OX crossover operator as PureGWO_NN, resulting in equivalent search capability. This result demonstrates that the gains of CLA components are not independently additive but arise from the complementary, orthogonal roles of exploration and exploitation: detection determines *when* to intervene, catastrophe determines *where* to intervene, and 2-opt determines *how to recover efficiently*. Each mechanism is therefore necessary but not sufficient alone: exploration without intensification cannot convert recovered diversity into high-quality solutions, while intensification without exploration cannot escape stagnation.

#### 4.2.8. Component Gain Chain

The complete performance gradient across all 7 variants can be summarized as follows:

$$\underset{297.3\%}{\underset{︸}{\mathrm{PureGWO}}}\overset{+\;\mathrm{NN}\;\mathrm{init}}{\to }\underset{75.2\%}{\underset{︸}{\mathrm{PureGWO\_NN}}}\overset{+\;\mathrm{CLA}\;(\mathrm{no}\;\mathrm{2-opt})}{\to }\underset{79.3\%}{\underset{︸}{\mathrm{No2opt}}}\overset{+\;\mathrm{2-opt}\;+\;\mathrm{CLA}}{\to }\underset{2.9\%}{\underset{︸}{\mathrm{Full}}}$$

Each transition’s gain and driving force are:

1. **PureGWO → PureGWO_NN (−222 pp)**: introducing one NN initial solution provides prior structural information; the gain stems from the OX crossover’s quality-edge propagation effect.
2. **PureGWO_NN → No2opt (+4 pp)**: the exploration-only CLA components match or slightly underperform the NN-initialized pure-GWO ceiling, confirming that exploration alone cannot substitute for the 2-opt intensification operator; the two mechanisms are complementary rather than additive.
3. **No2opt → Full (−76 pp)**: the synergy between 2-opt local search and the CLA detection–catastrophe–recovery system contributes the single largest gain span in the ablation study, with NoCLP (3.379%), NoMulti (3.317%), and NoTPR (3.110%) showing progressively decreasing degradation, indicating that CLP, multi-level catastrophe, and TPR each provide fine-tuning value in the near-optimal regime.

#### 4.2.9. Summary of Component Contributions

Synthesizing the 7-variant ablation and baseline results, the contribution hierarchy of CLA-GWO components can be categorized into three layers: **Layer 1 (Performance base): 2-opt + NN initialization.** 2-opt contributes approximately 76 pp of Sum_Gap gain (No2opt → Full), while NN initialization independently contributes 222 pp of Sum_Gap gain (PureGWO → PureGWO_NN); together they establish the algorithm’s performance floor. **Layer 2 (Diversity safeguard): CLP + multi-level catastrophe.** These two core components contribute ΔSum_Gap degradations of 0.476% and 0.414%, respectively, with stability degradation (standard deviation inflation of 1.8×–2.3×) that is even more pronounced than the mean degradation, confirming their critical role in maintaining search reliability. **Layer 3 (Auxiliary enhancement): TPR two-phase recovery.** Its independent contribution (ΔSum_Gap =+0.207%) is the smallest but consistently positive, primarily manifesting as structural diversity supplementation during post-catastrophe recovery on medium-scale instances, with scalable value in larger search landscapes.

Finally, the PureGWO baseline experiments confirm a subtle but important conclusion: component gains in metaheuristic algorithms are not linearly additive. The NN initialization and GWO crossover’s quality-edge propagation produce a superlinear gain (+55 pp in mean Gap), while the synergy between 2-opt and CLA depends on the simultaneous presence of both. This finding provides valuable insight into the design principles of hybrid metaheuristic algorithms.

**Contribution boundary: intensification vs. adaptive exploration regulation.** The ablation results clarify that 2-opt and CLA operate on orthogonal axes: 2-opt performs intensification (converting coarse search directions into high-quality solutions), while CLA performs *adaptive exploration regulation* (determining when and where to intervene, and calibrating sensitivity over time). Their contributions are therefore not directly comparable on the same metric. CLA’s independent regulatory value is evidenced by the stability degradation in Layer 2 variants (NoCLP: 1.8× std inflation; NoMulti: 2.3× std inflation) and by the sustained $\rho_{n}$ fluctuation on fl417 (Section 4.4), where CLA maintains productive exploration in regimes where 2-opt alone would converge prematurely.

### 4.3. Comparison with State-of-the-Art Algorithms

To rigorously evaluate the global optimization efficacy of CLA-GWO, extensive benchmark tests were conducted on 20 standard TSPLIB instances covering city scales from n=100 to n=417. Each instance was independently solved 30 times with different random seeds, and the best and mean Gap values were recorded. The SOTA comparison data for ABCSS, DSMO, DWHO, DDCS, AACO-LST, D-SFOA, PM, and GACS are drawn from their respective original publications. The comparison is organized into two groups: a primary group of widely referenced metaheuristics (ABCSS, DSMO, DWHO, GACS) and a secondary group of four recent metaheuristics (DDCS, AACO-LST, D-SFOA, and PM), together with GACS for cross-group referencing. The results of each group are reported below.

#### 4.3.1. Solution Quality on TSPLIB Instances

Table 16 compares the best optimization results obtained by CLA-GWO against four SOTA metaheuristic algorithms (ABCSS, DSMO, DWHO, and GACS) on all 20 TSPLIB instances.

The results demonstrate that CLA-GWO achieves the best or tied-best result among the compared algorithms in 19 of the 20 instances. CLA-GWO achieves a better best result than all four compared SOTA algorithms, including GACS, on 17 of the 19 comparable instances, with the margin over the strongest competitor reaching 0.94% (lin318) and 0.83% (d198). On the largest instance fl417 (n=417), CLA-GWO achieves a best value of 12,015, outperforming GACS (12060.22), DWHO (12192.53), DSMO (12218.98), and ABCSS (12356.44). The macro-averaged best Gap of CLA-GWO across all 20 instances is 0.13%, with 13 instances attaining the integer BKS exactly (best Gap of 0.00%) and the remaining seven instances within 1.30%.

A scale-dependent analysis reveals the following patterns:

**Small-scale (n≤136**, **9 instances).** CLA-GWO achieves near-optimal performance, attaining the integer BKS exactly on eight of the nine instances; the sole exception is pr136, whose best Gap is 0.02%. In particular, on lin105 CLA-GWO attains a best cost of 14,379 (Gap of 0.00%), tying DSMO at the exact BKS. On eil101, the floating-point best Gap of 1.78% disappears under the integer convention: CLA-GWO attains the BKS of 629 exactly (best Gap of 0.00%), as diagnosed in Section 4.5.

**Medium-scale (150≤n≤200**, **6 instances).** CLA-GWO maintains strong performance with an average best Gap of 0.03%. On kroA150, kroB150, pr152, and kroA200, CLA-GWO attains the BKS exactly (best Gap of 0.00%), outperforming all compared SOTA algorithms. On d198 and kroB200, CLA-GWO again leads all other compared algorithms, with best Gaps of 0.08% and 0.11%, respectively.

**Large-scale (n>200**, **5 instances).** CLA-GWO achieves an average best Gap of 0.49%, outperforming all compared SOTA algorithms on every instance and attaining the BKS on pr226. The performance gap widens with scale: on fl417, CLA-GWO outperforms the second-best algorithm (GACS) by 45.22 in tour cost; on lin318, by 402.31. This trend demonstrates that the catastrophe-driven closed-loop framework provides increasing competitive advantage as the search space dimensionality grows.

**Figure 5:**
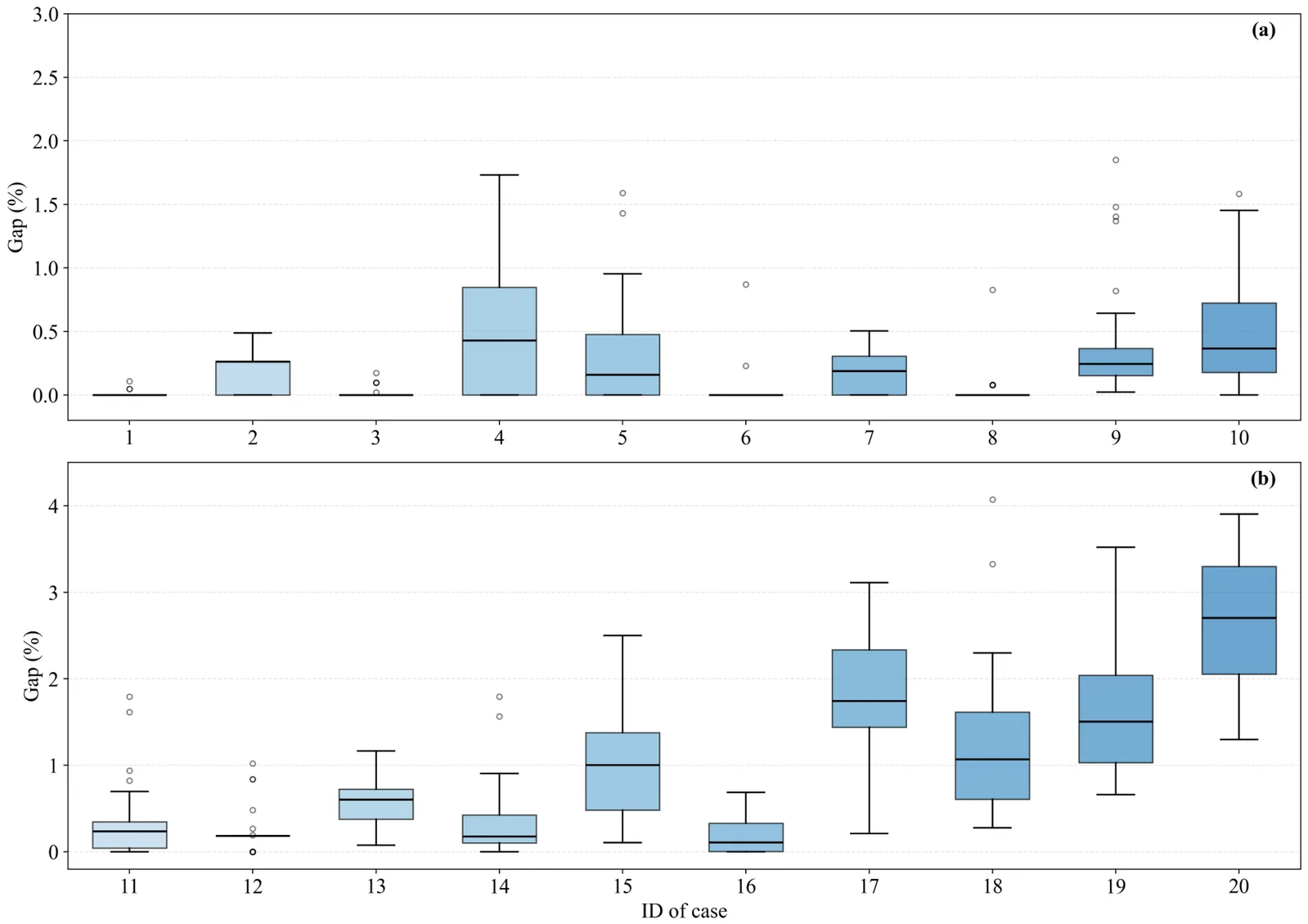
Gap distribution across 20 TSPLIB instances (30 independent runs each): (**a**) instances 1–10 (n=100–150); (**b**) instances 11–20 (n=150–417).

To complement the best-value comparison, Figure 5 presents the Gap distribution of CLA-GWO across all 20 instances over 30 independent runs, providing a comprehensive view of both solution quality and search stability. In each panel, boxes span the 25th–75th percentiles, the red line marks the median, whiskers extend to 1.5× the interquartile range (IQR), and circles denote outliers.

The boxplot reveals several key patterns:

**Small-scale stability.** On kroA100, kroC100, lin105, and pr124, the majority of runs (23–28 of 30) attain the BKS exactly (Gap of 0.00%), so the boxes collapse to thin lines at the zero baseline with only a few upper outliers (at most 0.87% on lin105). kroB100, pr107, and kroB150 also produce zero-Gap runs, with medians of 0.26%, 0.19%, and 0.24%, respectively, and lower whiskers reaching the zero baseline, confirming that these instances are solved to optimality in a substantial fraction of runs.

**Heterogeneous difficulty within the same scale.** Under the integer distance convention, eil101 no longer constitutes an outlier: its median Gap is 0.16%, and 12 of 30 runs attain the BKS (629) exactly, consistent with neighboring instances of comparable size. By contrast, rd100 and pr136 exhibit moderate dispersion, with medians of 0.43% and 0.24% and extreme runs reaching 1.73% and 1.85%, respectively, indicating that even within the same scale band, topological characteristics influence search difficulty.

**Scale-dependent dispersion growth.** As instance size increases from 150 to 417 cities, both the median Gap and the IQR grow systematically. In the medium range (150–200 cities), d198 and kroA200 maintain medians of 0.61% and 0.18%, while kroB200 rises to 1.00%. Among the large-scale instances with more than 200 cities, pr226 maintains a median of 0.11%, while gil262, pr299, lin318, and fl417 (with at least 262 cities) exhibit the highest medians (1.07–2.71%), with fl417’s entire box spanning 2.05–3.30%, the widest distribution among all instances. This progressive dispersion growth reflects the fundamental combinatorial complexity increase of TSP and is consistent with the convergence analysis in Section 4.4, where larger instances showed continued improvement through the final generation.

**Overall stability.** Across all 20 instances, the median Gap remains below 3%, with the majority (17 of 20) below 1.5%. No instance exhibits extreme dispersion: the whiskers remain within a bounded range, and the worst observed Gap across all 600 runs (20 instances × 30 runs) is 4.07% (pr299). This consistency confirms that CLA-GWO maintains stable convergence behavior across diverse topological configurations and problem scales, without catastrophic failures on any instance.

#### 4.3.2. Statistical Significance Analysis

To assess the statistical significance of the observed performance differences, a Friedman test was conducted on the 19 instances where the three independent baselines and CLA-GWO all have reported data (excluding kroC100 due to missing ABCSS and DSMO values); the subsequent pairwise Win/Tie/Loss analysis (Table 17) covers all instances where CLA-GWO and each competitor both have data (20 pairwise comparisons for DWHO and 19 for ABCSS and DSMO). GACS is a prior algorithm from the authors’ group; to keep this main ranking free of any conflict of interest, it is excluded here and is instead discussed separately in Section 4.5. The analysis ranks algorithms on each instance (1= best, 4= worst) and computes the average rank across all instances.

The Friedman test confirms a highly significant difference among the three independent baselines and CLA-GWO ($\chi^{2}\left(3\right)=37.20$, $p\approx 4.2\times 10^{-8}$). CLA-GWO achieves the best overall Average Rank of 1.026, substantially outperforming DWHO (2.500), DSMO (3.184), and ABCSS (3.289). The Nemenyi post-hoc test (CD =1.08 at α=0.05) confirms that CLA-GWO significantly outperforms DWHO, DSMO, and ABCSS. The pairwise Win/Tie/Loss analysis (Table 17) reinforces this conclusion: CLA-GWO never loses to any of the three independent baselines, winning all 20 comparable instances against DWHO, all 19 against ABCSS, and 18 of 19 against DSMO, with the sole non-win being a tie with DSMO on lin105. Because the results of the competing algorithms are cited from their original publications rather than re-implemented on the present platform, the Friedman ranking aggregates performance across heterogeneous experimental conditions; this residual environmental variance (hardware, compiler, and distance convention) should be borne in mind when interpreting the significance results below.

The sole non-win against the independent baselines is a tie with DSMO on lin105, where both algorithms attain the exact BKS of 14379 (best Gap of 0.00%). The comparison with GACS, the strongest competitor in the original primary-group comparison, is presented separately in Section 4.5 as a measure of progress within the authors’ own research line.

#### 4.3.3. Comparison with Recent Metaheuristics

To further validate competitiveness, a second group of four recent metaheuristics (DDCS, AACO-LST, D-SFOA, and PM), together with GACS for cross-group referencing, is included for comparison. Due to the limited overlap of test instances across these studies, the comparison is conducted on 7 shared TSPLIB instances (the 8-instance set from the original studies minus tsp225, which is not included in our test set). The results are presented in Table 18.

CLA-GWO achieves the best or tied-best result among the compared algorithms on 6 of 7 instances, attaining the integer BKS exactly on all seven instances; pr107 is the sole instance where CLA-GWO does not lead the raw-cost ranking, because two competitors report floating-point values (44,301.68) marginally below the integer BKS (44,303), a convention artifact discussed in Section 4.5.4, with particularly strong performance on kroA150 and kroB150 where it outperforms all five compared SOTA algorithms. On kroB100, CLA-GWO attains the BKS exactly with a best cost of 22,141, tying D-SFOA and outperforming all other compared algorithms. D-SFOA remains the strongest competitor in this group, achieving the BKS on 5 instances. However, CLA-GWO’s advantage becomes more pronounced at larger scales: on kroA150 (n=150), CLA-GWO achieves 26,524 (Gap of 0.00%), outperforming D-SFOA (26,687, Gap =0.62%) by a clear margin.

The results confirm that CLA-GWO remains competitive with the strongest recent metaheuristics, with distinct advantages at medium-to-large scales.

#### 4.3.4. Route Quality Visualization

**Figure 6:**
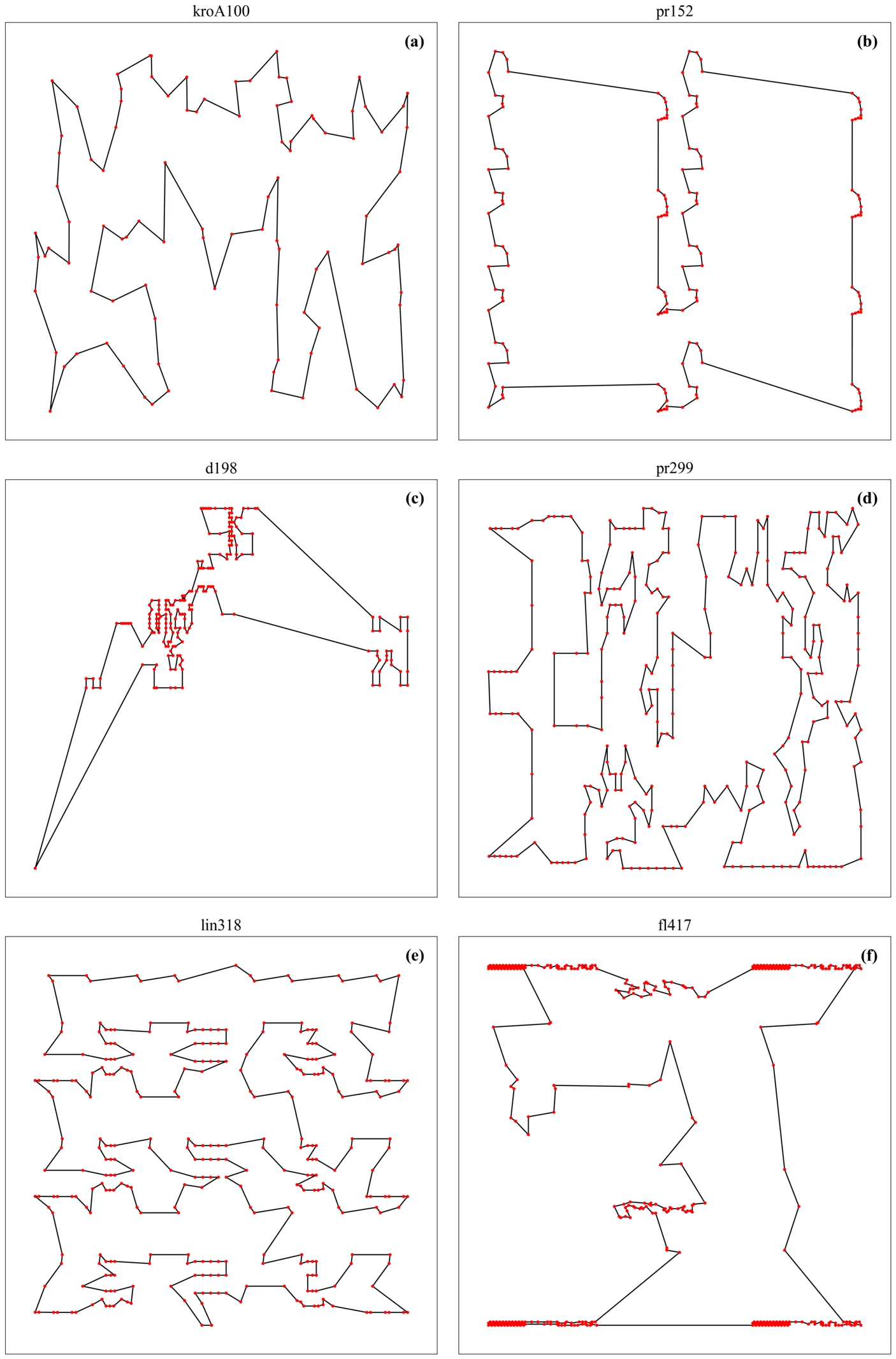
Best routes found by CLA-GWO on six TSPLIB instances: (**a**) kroA100; (**b**) pr152; (**c**) d198; (**d**) pr299; (**e**) lin318; (**f**) fl417.

Figure 6 presents the best routes found by CLA-GWO on six TSPLIB instances of increasing scale; each route is the best solution found across 30 independent runs per instance, except for fl417, for which a representative near-best tour is shown.

Across all six instances, the routes are smooth and coherent, with no significant edge crossings, a qualitative indicator of near-optimal structural quality. On kroA100 (Figure 6a), the route exhibits the characteristic “walnut-pattern” optimal structure on uniformly distributed points. On pr299 (Figure 6d), the elongated geographical distribution of Portugal (x-span ≈6677, y-span ≈2235) poses a challenge for population coverage, yet the algorithm achieves a mean Gap of 1.34%. On fl417 (Figure 6f), despite the highest city density and largest search space, the route maintains reasonable global structure without severe detours. As scale increases from 100 to 417 cities, the best Gap degrades gradually from 0.00% to 1.30%, but all routes preserve structural integrity, confirming the robustness of CLA-GWO across diverse topological configurations.

#### 4.3.5. Reference Comparison with Specialized TSP Solvers

To situate CLA-GWO relative to the strongest available TSP systems, Table 19 presents a reference comparison with three specialized solvers (Concorde [1], LKH [6], and EAX-GA [7]) on the seven TSPLIB instances for which specialized-solver results are available in [41]. As expected, all three specialized systems attain the BKS on every instance. CLA-GWO, as a general-purpose metaheuristic, attains the BKS exactly on six of the seven instances under the TSPLIB integer convention. The largest genuine residual gap is observed on lin318 (0.66%).

The objective of CLA-GWO is not to surpass these specialized systems in absolute optimality: Concorde is an exact solver that certifies optimality, LKH represents a mature form of variable-depth local search, and EAX-GA couples genetic recombination with dedicated local refinement. The comparison is nonetheless informative for the present study. The component ablation (Section 4.2) identified the 2-opt local search base as the principal performance driver of CLA-GWO, and the BKS-level accuracy of LKH on these instances illustrates the performance ceiling of precisely this algorithmic direction. In this sense, the reference comparison externally corroborates the ablation-based attribution: the closed-loop layers are not designed to outperform dedicated local search at its own game, but to enable a general-purpose framework to operate in the neighborhood of specialized solvers while maintaining population diversity throughout the search. The residual gap on lin318, the largest instance in this reference comparison, provides a quantified target for future work.

### 4.4. Convergence Behavior and Catastrophe Analysis

**Figure 7:**
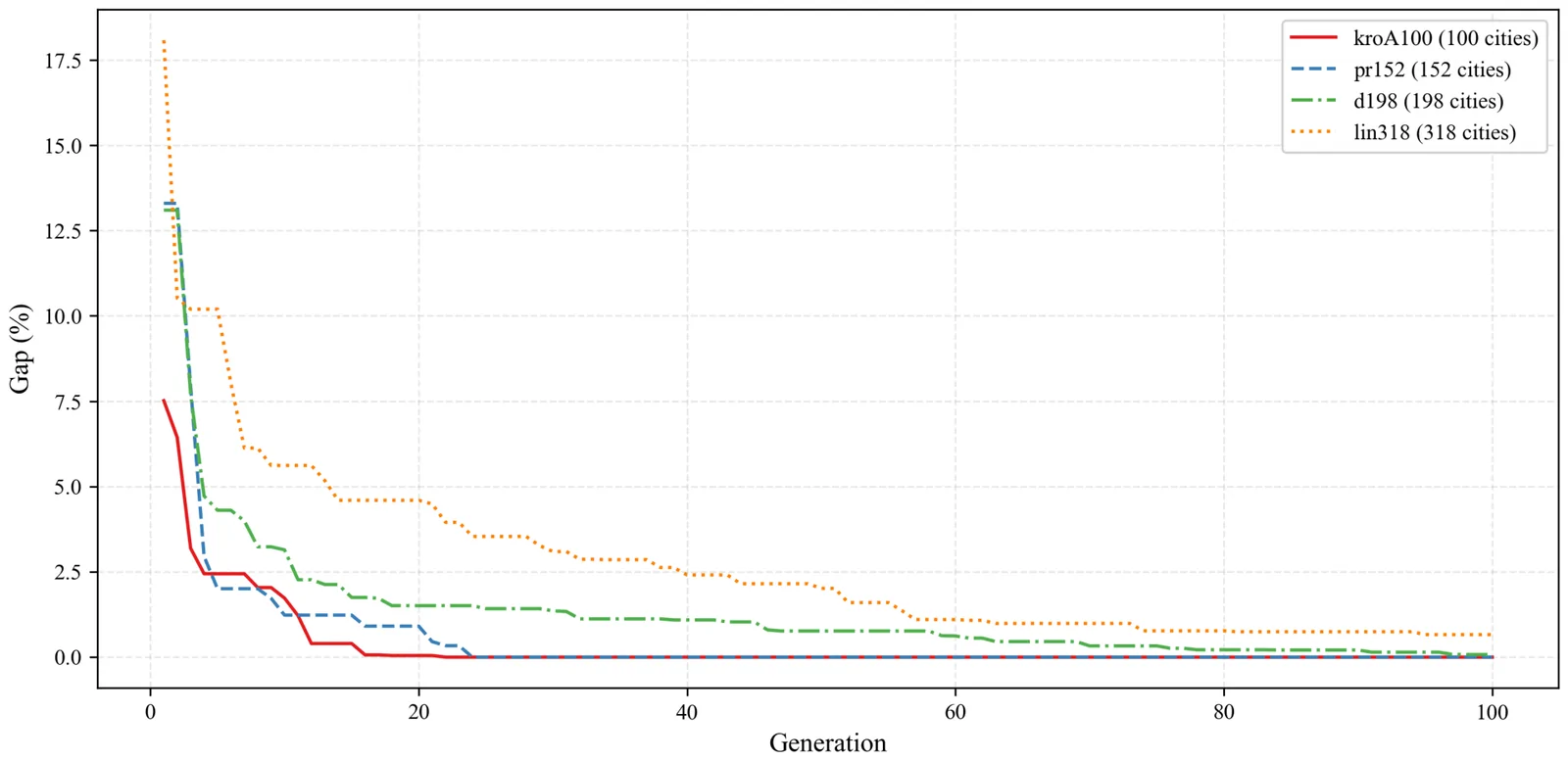
Convergence curves on four representative TSPLIB instances.

Figure 7 presents the convergence curves of CLA-GWO on four representative instances: kroA100 (n=100), pr152 (n=152), d198 (n=198), and lin318 (n=318), showing the Gap (%) as a function of generation; each curve is recorded from a single representative run per instance, with the seed matched to the SOTA comparison set.

All four curves exhibit a characteristic three-phase pattern: rapid descent, gradual deceleration, and final plateau. However, the convergence depth and rate differ significantly across scales. On kroA100, the initial Gap of 7.51% falls to 0.00% by generation 22, where it remains until termination. On pr152, convergence is similarly complete: from an initial Gap of 13.30%, the algorithm attains the BKS (Gap of 0.00%) by generation 24. In contrast, d198 and lin318 do not reach the BKS within the budget, continuing to improve through generations 98 and 95 with final Gaps of 0.08% and 0.66%, respectively. This indicates that for larger instances, 100 generations may be insufficient for full convergence, and the algorithm retains improvement potential at the termination point.

**Figure 8:**
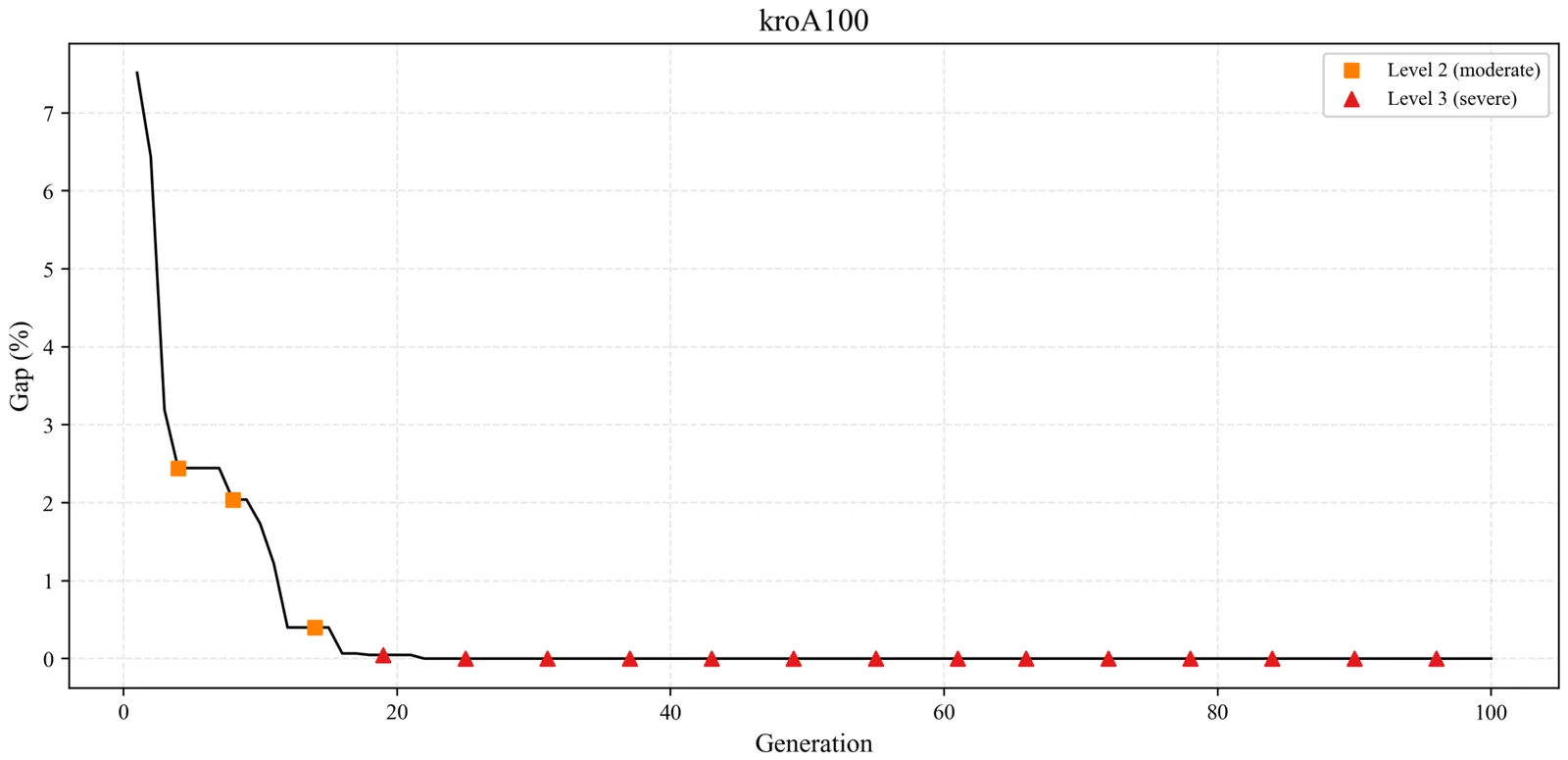
Catastrophe trigger records on kroA100 (n=100).

**Figure 9:**
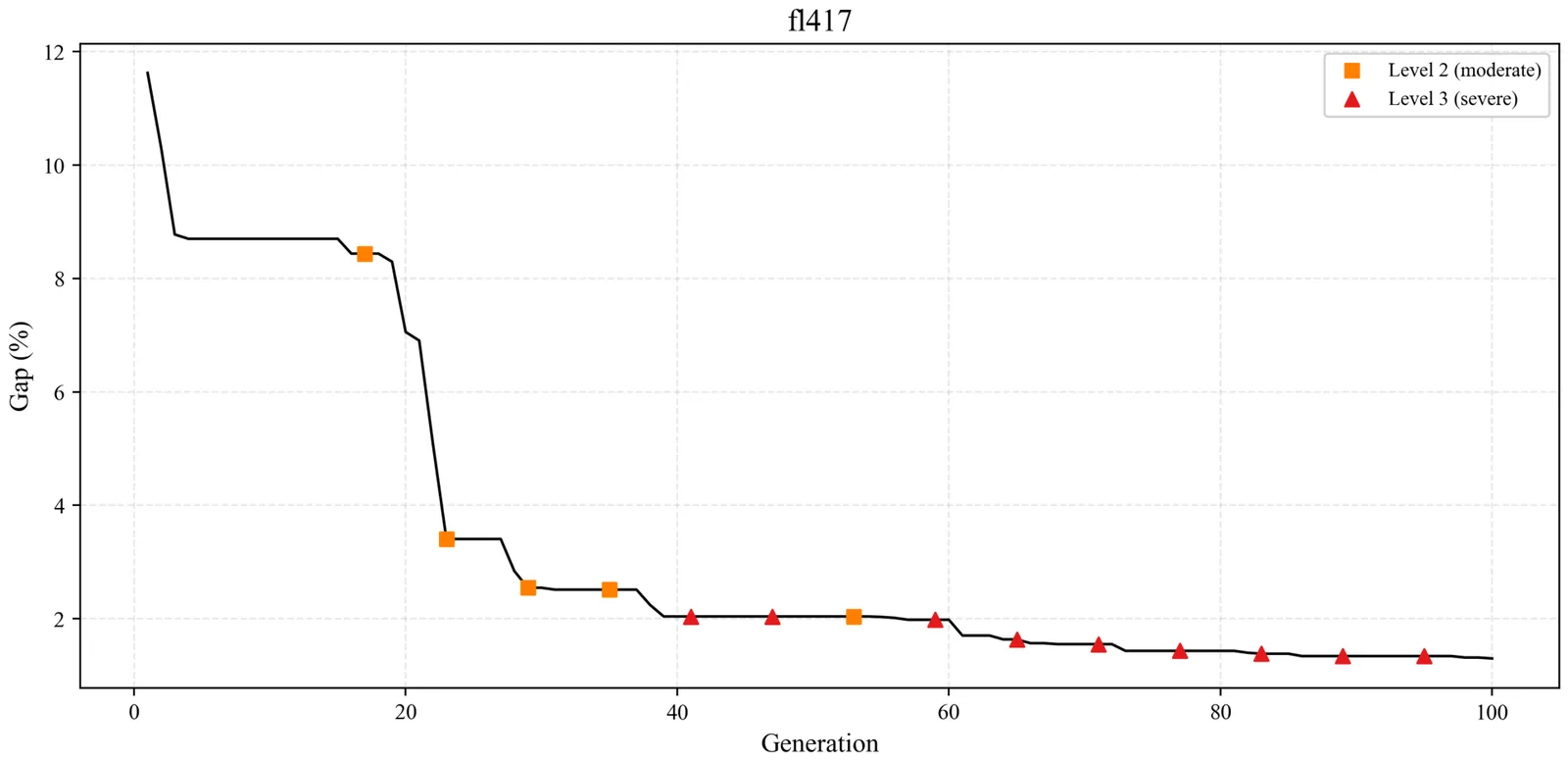
Catastrophe trigger records on fl417 (n=417).

Figure 8 and Figure 9 present the catastrophe trigger records on kroA100 and fl417, respectively, illustrating the scale-dependent behavior of the adaptive catastrophe mechanism.

On kroA100, the population homogenizes rapidly: the first catastrophe (level 1) triggers at generation 2 ($\rho_{n}=0.82$), followed by a level 3 catastrophe at generation 5 ($\rho_{n}=0.97$). From generation 25 onward, $\rho_{n}$ stabilizes at 1.0, indicating complete leadership-tier homogenization. At this stage, catastrophes no longer improve the solution but maintain search activity, preventing absolute stagnation.

On fl417, the behavior is markedly different: catastrophe levels alternate among levels 1–3 throughout the search, and $\rho_{n}$ fluctuates between 0.80 and 1.00 without prolonged fixation at 1.0. In this representative run, the best Gap continues to decrease through generation 98, reaching 1.30%. This pattern reveals that in larger search spaces, the diversity introduced by catastrophes is continuously absorbed by the population, sustaining productive exploration rather than mere activity maintenance.

This scale-dependent catastrophe behavior constitutes an emergent property of the adaptive framework: the same mechanism autonomously transitions between diversity maintenance mode (small-scale) and diversity creation mode (large-scale), without requiring manual parameter adjustment.

### 4.5. Discussion

The experimental results presented in Section 4.1, Section 4.2, Section 4.3 and Section 4.4 reveal several findings that warrant in-depth discussion, including the limited independent contribution of TPR, scale-dependent performance patterns, the non-additive nature of component gains, a distance convention artifact on eil101, the relation to the strongest baseline (GACS), and the algorithm’s limitations on large-scale instances.

#### 4.5.1. Limited Independent Contribution of TPR

The ablation analysis (Section 4.2) revealed that the NoTPR variant (ΔSum_Gap =+0.207%) performs comparably to the Full algorithm, particularly on rat195 where NoTPR even slightly outperforms Full (1.700% vs. 1.753%). This finding warrants a careful interpretation: TPR’s independent contribution to mean Gap improvement is the smallest among the ablated components, which is consistent with its role as an integrated sub-module of the catastrophe–recovery joint mechanism rather than a standalone contribution. The underlying reason is that the recovery 2-opt deep optimization in Phase 2 of TPR largely overlaps with the evolutionary and top-3 2-opt refinements already present in the GWO evolution layer and the post-catastrophe recovery. When 2-opt is active (as it is in all variants except No2opt), the marginal benefit of adding a separate ILS breadth search (Phase 1) is modest, because the OX crossover operator already provides structural recombination that partially covers the same search space. On rat195 specifically, the slight advantage of NoTPR over Full (1.700% vs. 1.753%) may be attributable to a budget reallocation effect: TPR’s Phase 1 ILS search consumes computational time that, in the NoTPR variant, is redirected to additional GWO evolution generations, yielding marginally better exploration on this particular instance’s search landscape. However, retaining TPR is justified on two grounds: first, its contribution is consistently positive across medium-scale instances (rd100: +0.148 pp, kroB150: +0.112 pp), confirming a real if small effect; second, the ILS breadth search with Metropolis acceptance provides a mechanism for escaping local optima that 2-opt alone cannot achieve, and this mechanism becomes increasingly valuable in larger or more rugged search landscapes.

#### 4.5.2. Scale-Dependent Performance and Catastrophe Mode Transition

The convergence and catastrophe analysis (Section 4.4) revealed a striking scale-dependent behavioral pattern: on small-scale instances (n≤136), the population homogenizes rapidly and catastrophes shift to “maintenance mode” (level 3, $\rho_{n}=1.0$), primarily preventing stagnation rather than creating new diversity; on large-scale instances (n>200), catastrophes alternate across levels 1–3 with $\rho_{n}$ fluctuating between 0.80 and 1.00, sustaining productive exploration throughout the search. This emergent mode transition, achieved without any manual parameter adjustment, constitutes one of the most distinctive properties of the CLA-GWO framework and is a direct consequence of the self-learning threshold adjustment mechanism (Section 2.5.1), which autonomously calibrates detection sensitivity based on historical catastrophe effectiveness.

Despite this adaptive behavior, the performance gap between small and large scales remains substantial: the average mean Gap increases from 0.20% (small) to 1.50% (large), and on fl417 (n=417) the mean Gap reaches 2.64%. The convergence curves (Figure 7) show that d198 and lin318 continue improving through generation 98, suggesting that 100 generations is insufficient for full convergence on large instances. This indicates that the primary bottleneck on large-scale TSP is not the algorithm’s search capability but rather the computational budget allocated per instance. Increasing the generation limit for large instances, or implementing an adaptive termination criterion based on improvement rate, is a promising direction.

#### 4.5.3. Non-Additive Component Synergy

The PureGWO baseline experiments (Section 4.2) demonstrated that component gains in CLA-GWO are not linearly additive. The most striking evidence is the finding that PureGWO_NN (75.2%) and No2opt (79.3%) achieve nearly identical performance: isolated from the 2-opt intensification operator, the CLA exploration mechanisms match a pure GWO initialized with a single NN solution. This demonstrates the orthogonal division of labor between exploration and exploitation: the detection mechanism determines *when* to intervene, the catastrophe mechanism determines *where* to intervene, and 2-opt determines *how to recover efficiently*. Neither the exploration loop nor the intensification operator is sufficient alone; their combination, rather than the superiority of any single component, yields the full performance. The reference comparison with specialized solvers (Section 4.3.5) externally corroborates this attribution: LKH, the mature form of variable-depth local search, attains the BKS on all seven shared instances, whereas CLA-GWO attains the BKS on six of them. Conversely, the NN initialization and GWO crossover produce a superlinear gain (+55 pp in mean Gap) through quality-edge propagation, far exceeding what either component could achieve alone. These findings have broader implications for the design of hybrid metaheuristic algorithms: component synergy is not guaranteed by simple juxtaposition but requires careful alignment of complementary roles, intensification (2-opt) and adaptive exploration regulation (CLA), rather than competing on the same axis.

#### 4.5.4. Sensitivity of the Reported Gap to the Distance Convention

A tour’s integer cost is a deterministic function of the tour and the TSPLIB rounding rule, independent of the convention under which the tour was found; the reported Gap difference between the two conventions therefore decomposes into a metric effect (rounding the same tour) and a search effect (the two objectives converging to different tours). For eil101, the metric effect dominates: the floating-point best tours have a mean cost of about 641.69 but re-evaluate to a mean integer cost of about 630.10, with the best run reaching 629, exactly the BKS.

The effect is instance-dependent rather than uniform. Table 20 summarizes the means over 10 paired runs with identical seeds: the metric effect is about 1.84 percentage points on eil101 but only about 0.31 and 0.33 pp on gil262 and fl417, respectively. After correcting for this metric, gil262 and fl417 retain mean Gaps of 1.58% and 2.54%, confirming that their difficulty is genuine rather than a convention artifact.

Accordingly, all absolute Gap values reported against the BKS in Section 4.3 and Section 4.4 use the TSPLIB integer convention defined in Section 3.2.3.

#### 4.5.5. Relation to the Strongest Baseline (GACS)

As declared in Section 3.4 and in the note to Table 16, GACS [41] is a prior algorithm developed by the authors’ research group, and its comparison data are cited directly from the original publication rather than re-implemented. Among the compared algorithms, GACS is the strongest competitor, and the performance margin over it is accordingly the narrowest. Nevertheless, CLA-GWO remains ahead on every aggregate measure: in the direct pairwise comparison it wins 19 of the 20 comparable instances with one loss (on pr107), and its advantage widens with problem scale, reaching 45.22 in tour cost on fl417. For transparency, the comparison against GACS should be interpreted as a measure of progress within the authors’ own research line, whereas the comparisons against the remaining baselines provide an independent external benchmark.

#### 4.5.6. Runtime Scalability

**Figure 10:**
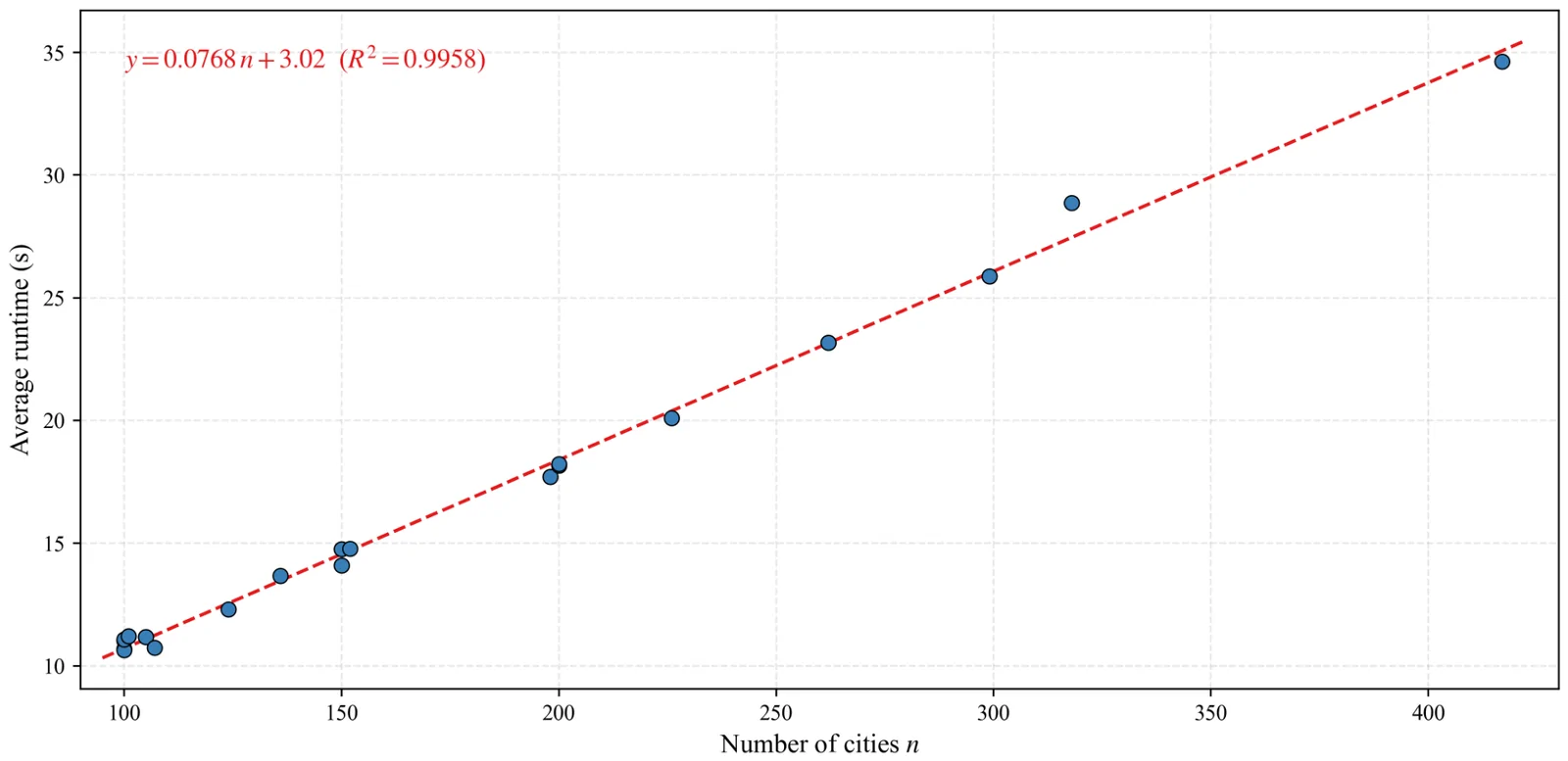
Runtime scalability of CLA-GWO on 20 TSPLIB instances (n=100–417): average runtime over 30 independent runs per instance; the red dashed line denotes the linear fit.

Figure 10 presents the runtime scalability of CLA-GWO across all 20 benchmark instances. The average runtime increases smoothly from approximately 10 s (n=100) to 35 s (n=417). Both linear and power-law models were fitted to the measurements: the linear fit y=0.0768n+3.02 attains $R^{2}=0.9958$, whereas the power-law fit $y=0.2546\;n^{0.810}$ attains $R^{2}=0.9928$; the linear model is accordingly retained as the more accurate description, indicating an incremental cost of only ≈0.08 s per additional city. This near-linear growth is consistent with the per-generation complexity $O(N\cdot n\cdot c_{\mathrm{cand}})$ under fixed generation budgets. The power-law exponent (0.810<1) further confirms slightly sublinear growth with no rapid escalation up to n=417.

#### 4.5.7. Limitations

This study has several limitations that should be acknowledged: (i) The parameter optimization process (TPE + OAT) requires 4000 algorithm invocations for the coarse search alone, which may be prohibitively expensive for practitioners without dedicated computing resources; a simplified parameter recommendation protocol could be developed for deployment scenarios. (ii) Performance on large-scale instances (n>300) can still be improved, partly due to insufficient generation budgets; adaptive generation allocation could address this issue. (iii) The algorithm is evaluated exclusively on symmetric TSP instances; its applicability to asymmetric TSP (ATSP), multiple traveling salesman problems (mTSP), or vehicle routing problems (VRP) remains untested. (iv) The SOTA comparison data are drawn from original publications rather than re-implemented under a unified framework; differences in hardware, implementation language, compiler, and random-seed protocol introduce uncontrollable environmental variance, so the reported rankings should be read as indicative of competitive standing rather than as proof of absolute superiority. (v) The dual distance convention (Section 3.2.3) requires care when comparing Gap values across subsections, because absolute and relative Gap values are not directly interchangeable. (vi) The maximum test scale is n=417; while runtime scales near-linearly up to this point (Figure 10, $R^{2}=0.996$), extrapolation to ultra-large-scale TSP (n>1000) remains unverified. Per-generation cost grows linearly with *n*, but the number of generations required for convergence may grow super-linearly, making adaptive generation allocation and parallel implementation critical for scalability extension (see Section 5). (vii) No formal convergence or diversity-preservation guarantee is established; the discrete, non-differentiable, and adaptive nature of the closed loop makes such an analysis intractable, so the results are validated empirically.

## 5. Conclusions

This paper proposed the Closed-Loop Adaptive Grey Wolf Optimizer (CLA-GWO), a five-layer closed-loop adaptive metaheuristic designed to address the fundamental diversity loss problem of the standard Grey Wolf Optimizer when solving the Traveling Salesman Problem. Coupling discrete GWO evolution, dual-threshold diversity detection, a three-level adaptive catastrophe mechanism, two-phase recovery, and self-learning feedback into a single loop yields a complete “detection–catastrophe–recovery–learning” cycle that autonomously perceives population homogenization and restores diversity without manual intervention, in concert with the 2-opt local search base.

Comprehensive experiments on 20 TSPLIB instances demonstrated that CLA-GWO achieves the best or tied-best result on 19 of the 20 instances under the adopted benchmark protocol against eight state-of-the-art algorithms, with a Friedman test confirming a highly significant overall difference among the three independent baselines and CLA-GWO ($\chi^{2}\left(3\right)=37.20$, $p\approx 4.2\times 10^{-8}$). Parameter sensitivity analysis, component ablation, and convergence analysis further showed that performance is principally driven by the 2-opt local search and nearest-neighbor initialization, while the catastrophe and self-learning layers safeguard diversity and enable a scale-adaptive transition between diversity maintenance and creation without manual parameter adjustment.

Despite these results, several limitations remain (as detailed in Section 4.5), including the computational cost of parameter optimization, the unverified scalability beyond n=417, the restriction to symmetric TSP, and the reliance on publication-reported rather than re-implemented baselines.

Future work will pursue three directions: (1) adaptive computational-efficiency mechanisms, combining dynamic generation allocation with on-demand TPR activation to reduce overhead; (2) extension of the five-layer closed-loop framework to related combinatorial problems (VRP, flow-shop scheduling) and TSP variants (ATSP, clustered TSP); and (3) parallel implementation to reduce wall-clock time and support ultra-large-scale instances.

More broadly, the five-layer closed-loop framework, comprising diversity detection, adaptive intervention, structured recovery, and self-learning calibration, is not limited to GWO or TSP. The core principle of the PDIRL cycle suggests a transferable design pattern for other population-based metaheuristics facing premature convergence, although its current demonstration is limited to symmetric TSP instances.

## Figures and Tables

**Table 1:** Case 4 benchmark instances for parameter optimization and ablation.

Instance	*n*	BKS	Time Budget (s)	Scale
berlin52	52	7544	3	Small
rd100	100	7910	6	Medium
kroB150	150	26,130	12	Medium
rat195	195	2323	18	Large

Note: the scale labels are relative rankings within this four-instance set and do not follow the size tiers defined for the Case 20 benchmark (Section 3.1.2).

**Table 2:** Case 20 benchmark instances for SOTA comparison.

ID	Instance	BKS	Scale	ID	Instance	BKS	Scale
1	kroA100	21,282	S	2	kroB100	22,141	S
3	kroC100	20,749	S	4	rd100	7910	S
5	eil101	629	S	6	lin105	14,379	S
7	pr107	44,303	S	8	pr124	59,030	S
9	pr136	96,772	S	10	kroA150	26,524	M
11	kroB150	26,130	M	12	pr152	73,682	M
13	d198	15,780	M	14	kroA200	29,368	M
15	kroB200	29,437	M	16	pr226	80,369	L
17	gil262	2378	L	18	pr299	48,191	L
19	lin318	42,029	L	20	fl417	11,861	L

**Table 3:** Complete parameter configuration of CLA-GWO. ^†^ denotes tunable parameters optimized in Section 4.1.

Symbol	Code Key	Value	Description
*GWO Evolution*			
N ^†^	pop_size	20	Population size
$I_{\mathrm{gwo}}$	max_imp_gwo	20	2-opt budget for ordinary wolves
$I_{\mathrm{top3}}$	max_imp_top3	30	2-opt budget for leading wolves
—	init_nn_random	True	One NN heuristic solution + (N−1) random permutations
—	init_use_2opt	False	Disable 2-opt at initialization
$Gen_{\mathrm{ref}}$	gen_ref	200	Convergence factor *a* decay reference period (fixed at twice the generation budget)
*Detection*			
$\tau_{n}$ ^†^	tau_narrow	0.800	Narrow-scope detection threshold
$\tau_{w}$ ^†^	tau_wide	0.645	Wide-scope detection threshold
*K*	k_wide	10	Wide-scope wolf count
*Catastrophe*			
$r_{c}^{\left(1\right)}$	cat_ratio_1	0.20	level-1 wolf selection ratio
$r_{c}^{\left(2\right)}$	cat_ratio_2	0.40	level-2 wolf selection ratio
$r_{c}^{\left(3\right)}$	cat_ratio_3	0.60	level-3 wolf selection ratio
$r_{d}^{\left(1\right)}$	dest_ratio_1	0.05	level-1 segment disruption ratio
$r_{d}^{\left(2\right)}$	dest_ratio_2	0.15	level-2 segment disruption ratio
$r_{d}^{\left(3\right)}$	dest_ratio_3	0.30	level-3 segment disruption ratio
*TPR Recovery*			
$I_{\max}$ ^†^	max_imp_2opt	20	2-opt maximum improvement budget
$k_{1}$ ^†^	ils_k1	1	ILS breadth search rounds
$k_{2}$ ^†^	opt_k2	5	2-opt depth multiplier
*Self-Learning*			
η ^†^	eta	0.20	Self-learning rate
*H*	n_history	3	History window length
$L_{\max}$ ^†^	max_clp	5	Maximum lockout period
*Component Switches*			
—	use_2opt	True	Enable 2-opt local search
—	use_tpr	True	Enable TPR two-phase recovery
—	use_dynamic_clp	True	Enable dynamic lockout period
—	use_multi_level	True	Enable multi-level catastrophe

**Table 4:** Computational environment and comparison methodology.

Algorithm	Source	Platform	Data Origin
CLA-GWO	This work	i7-7700K, Python 3.13	Self-experiment
ABCSS	[42] (2019)	Original pub. ^a^	Cited
DSMO	[43] (2020)	Original pub. ^a^	Cited
DWHO	[44] (2024)	Original pub. ^a^	Cited
GACS	[41] (2026)	Original pub. ^a^	Cited
DDCS	[45] (2025)	Original pub. ^a^	Cited
AACO-LST	[46] (2023)	Original pub. ^a^	Cited
D-SFOA	[47] (2025)	Original pub. ^a^	Cited
PM	[12] (2024)	Original pub. ^a^	Cited

^a^ Original publication; hardware and software details as reported therein.

**Table 5:** Per-instance CLA-GWO runtime by instance scale (30 runs per instance).

Scale	*n* Range	Avg. Time (s)	Range (s)
S (n≤136)	100–136	11–14	10.6–13.7
M (150≤n≤200)	150–200	14–18	14.1–18.2
L (226≤n≤417)	226–417	20–35	20.1–34.6

**Table 6:** Overall statistics of the TPE hyperparameter search.

Metric	Value	Unit
Number of trials	100	—
Runs per trial per instance	10	—
Best Gap	0.5557	%
Worst Gap	1.0560	%
Mean Gap ± Std	0.7645±0.1012	%
Median Gap	0.7527	%
Q25–Q75	0.6949–0.8350	%

**Table 7:** Parameter sensitivity analysis: Top-10 vs. Bottom-10 comparison.

Parameter	Top-10 Mean	Bottom-10 Mean	Diff.	Direction
*N*	20.0	38.0	−18.0	Lower → Better
$L_{\max}$	11.0	9.0	+2.0	Higher → Better
$I_{\max}$	36.0	33.0	+3.0	Higher → Better
$\tau_{n}$	0.8106	0.8472	−0.0366	Lower → Better
η	0.1930	0.1400	+0.0530	—
$k_{2}$	4.30	4.80	−0.50	—
$k_{1}$	1.6	2.6	−1.0	—
$\tau_{w}$	0.6987	0.6848	+0.0139	—

**Table 8:** Optimal parameter configuration (Trial 65, overall mean Gap =0.5557%).

Parameter	Value	Parameter	Value
*GWO Evolution*			
*N* (population size)	20	$I_{\mathrm{gwo}}$	20
$I_{\mathrm{top3}}$	30	NN random start	True
Init 2-opt	False		
*Detection*			
$\tau_{n}$ (narrow threshold)	0.800	$\tau_{w}$ (wide threshold)	0.645
*K* (wide-scope wolves)	10		
*Catastrophe*			
$r_{c}^{\left(1\right)}$	0.20	$r_{d}^{\left(1\right)}$	0.05
$r_{c}^{\left(2\right)}$	0.40	$r_{d}^{\left(2\right)}$	0.15
$r_{c}^{\left(3\right)}$	0.60	$r_{d}^{\left(3\right)}$	0.30
*TPR*			
$I_{\max}$ (2-opt budget)	20	$k_{1}$ (ILS breadth)	1
$k_{2}$ (2-opt depth)	5		
*Self-Learning*			
η (learning rate)	0.20	*H* (history window)	3
$L_{\max}$ (max lockout)	5		
*Component Switches*			
2-opt enabled	True	TPR enabled	True
Dynamic CLP	True	Multi-level catastrophe	True

**Table 9:** OAT sensitivity scan results. SR = success rate (Gap < 1%).

**Population Size** ***N***			**2-Opt Budget** $I_{\max}$		
**Value**	**Gap (%)**	**SR** **(%)**	**Value**	**Gap (%)**	**SR (%)**
15	0.836	60	10	0.896	62
20	0.631	75	20	0.637	75
25	0.838	60	30	0.895	62
30	0.745	67	40	0.977	57
35	0.852	57	50	0.827	60
40	0.924	52	60	0.852	65
45	0.930	57			
50	1.019	60			
**Lockout period** $L_{\max}$			**Detection threshold** $\tau_{n}$		
**Value**	**Gap (%)**	**SR (%)**	**Value**	**Gap (%)**	**SR (%)**
3	0.787	62	0.75	0.700	67
5	0.635	75	0.77	0.730	67
7	0.667	65	0.79	0.658	72
9	0.752	62	0.81	0.614	75
11	0.722	65	0.83	0.619	75
13	0.702	70	0.85	0.683	75
15	0.699	65	0.87	0.721	72
18	0.676	67	0.89	0.709	70
20	0.676	67			

**Table 10:** 30-run independent validation of the optimal configuration. SR = success rate (Gap < 1%).

Instance (*n*)	Mean Gap (%)	Std (%)	Best (%)	Worst (%)	SR (%)
berlin52 (52)	0.005	<0.001	0.005	0.005	100
rd100 (100)	0.602	0.590	0.005	1.756	77
kroB150 (150)	0.543	0.323	0.003	1.365	90
rat195 (195)	1.753	0.587	0.816	3.133	13
Sum of Gaps	2.903	—	—	—	70

**Table 11:** Candidate list size determination: mean Gap (%) on three representative instances.

$c_{\mathrm{cand}}$	kroA100 (n=100)	kroB150 (n=150)	rat195 (n=195)	Avg. Time (s)
5	0.000	0.209	1.584	15.9
10	0.000	0.423	1.502	15.6
15	0.000	0.379	1.403	15.1
20	0.000	0.184	1.412	14.9

**Table 12:** 2-opt trigger threshold determination: mean Gap (%) on three instances.

Threshold	kroA100	kroB150	rat195	Avg. Time (s)
1.5	0.005	0.129	1.412	14.5
1.8	0.000	0.411	1.356	14.8
2.0	0.011	0.243	1.227	16.4
2.5	0.005	0.321	1.580	15.6

**Table 13:** $\tau_{\mathrm{eff}}$ truncation bounds sensitivity: mean Gap (%) on three instances.

Bounds	kroA100	kroB150	rat195	Avg. Time (s)
[0.70,0.98]	0.000	0.413	1.352	16.4
[0.75,0.95]	0.016	0.403	1.068	15.8
[0.75,0.98]	0.009	0.354	1.115	16.5
[0.80,0.98]	0.009	0.419	1.188	16.1

**Table 14:** Component ablation and pure-GWO baseline comparison (Gap, %). ΔSum_Gap denotes the Sum_Gap difference relative to Full (positive = degradation).

Variant	berlin52	rd100	kroB150	rat195	Sum_Gap	ΔSum_Gap
Full	0.005	0.602	0.543	1.753	2.903	—
NoTPR	0.005	0.750	0.655	1.700	3.110	+0.207
NoCLP	0.005	0.749	0.781	1.844	3.379	+0.476
NoMulti	0.005	0.917	0.644	1.751	3.317	+0.414
No2opt	14.870	21.353	26.132	16.910	79.265	+76.362
PureGWO_NN	13.708	20.969	23.510	17.059	75.246	+72.343
PureGWO	35.745	69.051	96.429	96.107	297.333	+294.430

**Table 15:** Paired Wilcoxon signed-rank tests (two-tailed, 20 paired runs) of Full versus each ablation variant. *** p<0.001; ns, not significant.

Variant	berlin52	rd100	kroB150	rat195
NoTPR	ns	***	***	ns
NoCLP	ns	***	***	ns
NoMulti	ns	***	***	ns
No2opt	***	***	***	***
PureGWO	***	***	***	***
PureGWO_NN	***	***	***	***

**Table 16:** Comparison of best optimization results on 20 TSPLIB instances. “/” denotes data unavailable in the original publication. GACS [41] is a prior algorithm developed by the authors’ research group.

Instance	*n*	BKS	ABCSS	DSMO	DWHO	GACS	CLA-GWO
kroA100	100	21,282	21,299	21,298.21	21,285.44	21,285.44	21,282
kroB100	100	22,141	22,229.71	22,308	22,244.83	22,193.65	22,141
kroC100	100	20,749	/	/	20,750.76	20,750.76	20,749
rd100	100	7910	7944.32	8041.30	7917.38	7910.40	7910
eil101	101	629	646.05	648.66	647.82	642.08	629
lin105	105	14,379	14,406.12	14,379	14,472.86	14,383	14,379
pr107	107	44,303	44,525.68	44,385.86	44,480.74	44,301.68	44,303
pr124	124	59,030	59,030.74	60,285.21	59,030.74	59,030.74	59,030
pr136	136	96,772	97,853.91	97,538.68	98,418.28	96,920.38	96,794
kroA150	150	26,524	26,981.98	27,591.44	27,158.73	26,603.89	26,524
kroB150	150	26,130	26,760.79	26,601.94	26,629.85	26,194.60	26,130
pr152	152	73,682	74,337.62	74,243.91	73,821.25	73,683.64	73,682
d198	198	15,780	16,270.22	15,978.13	15,941.50	15,924.18	15,792
kroA200	200	29,368	30,701.86	30,481.35	30,290.53	29,430.38	29,368
kroB200	200	29,437	31,508.85	30,716.50	30,340.10	29,639.15	29,468
pr226	226	80,369	82,266	83,587.98	80,733.86	80,374.25	80,369
gil262	262	2378	2526.99	2543.15	2460.15	2400.42	2383
pr299	299	48,191	50,265.88	50,579.82	50,055.86	48,491.56	48,325
lin318	318	42,029	45,135.50	44,118.66	43,791.51	42,709.31	42,307
fl417	417	11,861	12,356.44	12,218.98	12,192.53	12,060.22	12,015

**Table 17:** Pairwise Win/Tie/Loss comparison of CLA-GWO against three independent SOTA algorithms (best values, on all instances where both algorithms of each pair have reported data).

CLA-GWO vs.	Win	Tie	Loss
ABCSS	19	0	0
DSMO	18	1	0
DWHO	20	0	0

**Table 18:** Comparison with recent metaheuristic algorithms on shared TSPLIB instances (best values).

Instance	BKS	DDCS	AACO-LST	D-SFOA	PM	GACS	CLA-GWO
kroA100	21,282	21,282	21,285.44	21,282	21,575.98	21,285.44	21,282
kroB100	22,141	22,199	22,263.98	22,141	22,486.55	22,193.65	22,141
kroC100	20,749	20,749	20,750.76	20,749	21,103.32	20,750.76	20,749
lin105	14,379	14,379	14,383	14,379	14,501.10	14,383	14,379
pr107	44,303	44,303	44,301.68	44,303	44,781.22	44,301.68	44,303
kroA150	26,524	26,773	26,787.78	26,687	27,801.51	26,603.89	26,524
kroB150	26,130	26,303	26,156.38	26,233	27,133.53	26,194.60	26,130

**Table 19:** Reference comparison with specialized TSP solvers on the seven TSPLIB instances for which specialized-solver results are available in [41]. Concorde, LKH, and EAX-GA results are as compiled in [41]; all three solvers attain the BKS on every instance. CLA-GWO values are the best results from Table 16.

Instance	*n*	BKS	Concorde	LKH	EAX-GA	CLA-GWO	Gap (%)
kroA100	100	21,282	21,282	21,282	21,282	21,282	0.000
kroC100	100	20,749	20,749	20,749	20,749	20,749	0.000
rd100	100	7910	7910	7910	7910	7910	0.000
eil101	101	629	629	629	629	629	0.000
kroA150	150	26,524	26,524	26,524	26,524	26,524	0.000
kroA200	200	29,368	29,368	29,368	29,368	29,368	0.000
lin318	318	42,029	42,029	42,029	42,029	42,307	0.661

**Table 20:** Effect of the distance convention on the reported Gap (10 paired runs per instance, identical seeds).

Instance	BKS	Float Gap (Mean, %)	Integer Gap (Same Tour, Mean, %)	Metric Effect (pp)
eil101	629	2.02	0.18	1.84
gil262	2378	1.89	1.58	0.31
fl417	11,861	2.86	2.54	0.33

## Data Availability

The data and statistical test codes presented in this study are available from the corresponding author upon reasonable request. All randomized experiments are controlled by fixed random-seed settings: the SOTA comparison uses 30 independent runs per instance with distinct seeds, the component ablation and the eil101 diagnosis use 20 independent runs, and each parameter level of the sensitivity scans is evaluated over 10 independent runs. The per-run best, mean, and worst values reported in this paper were all recorded under these fixed seed settings, so that every statistical result can be traced back to, and reproduced from, the stored run-level data.

## Abbreviations

The following abbreviations are used in this manuscript:

**Abbreviations**	**Full Name**
2-opt	2-opt Local Search
AACO-LST	Adaptive Ant Colony Optimization with Local Search Tabu
ABCSS	Artificial Bee Colony with Scatter Search
ACO	Ant Colony Optimization
ATSP	Asymmetric Traveling Salesman Problem
BKS	Best Known Solution
CD	Critical Difference
CLA-GWO	Closed-Loop Adaptive Grey Wolf Optimizer
CLP	Catastrophe Lockout Period
D-SFOA	Discrete Salp Fruit-fly Optimization Algorithm
DDCS	Discrete Differential Cuckoo Search
DSMO	Discrete Spider Monkey Optimization
DWHO	Discrete Wild Horse Optimizer
EAX-GA	Edge-Assembly Crossover Genetic Algorithm
GACS	Globally Adaptive Ant Colony System
GWO	Grey Wolf Optimizer
ILS	Iterated Local Search
IQR	Interquartile Range
LKH	Lin–Kernighan–Helsgaun (heuristic)
ML	Machine Learning
mTSP	Multiple Traveling Salesman Problem
NN	Nearest Neighbor
OAT	One-Factor-At-a-Time
OX	Order Crossover
PDIRL	Perceive–Decide–Intervene–Recover–Learn
PM	Proposed Method (ant-system-based collective intelligence method of Kiran
	and Beskirli)
RL	Reinforcement Learning
SOTA	State-of-the-Art
SR	Success Rate
Sum_Gap	Sum of Gaps
TPE	Tree-structured Parzen Estimator
TPR	Two-Phase Recovery
TSP	Traveling Salesman Problem
VRP	Vehicle Routing Problem
